# The effect of dexmedetomidine in mechanically ventilated patients with sepsis and septic shock: a meta-analysis of randomized controlled trials

**DOI:** 10.1080/07853890.2026.2643971

**Published:** 2026-03-17

**Authors:** Lin Chen, Weibing Wu, Yunxiang Chen, Minfeng Tong, Binbin Ren, Kai Zhang

**Affiliations:** a Department of Neurosurgery Intensive Care Unit, Department of Neurosurgery, Affiliated Jinhua Hospital, Zhejiang University School of Medicine, Jinhua, China; b Department of Critical Care Medicine, Zhejiang Qingyuan People’s Hospital, Qingyuan, China; c Department of Neurosurgery, Affiliated Jinhua Hospital, Zhejiang University School of Medicine, Jinhua, China; d Department of Infectious Diseases, Affiliated Jinhua Hospital, Zhejiang University School of Medicine, Jinhua, China; e Department of Critical Care Medicine, Second Affiliated Hospital, Zhejiang University School of Medicine, Hangzhou, China

**Keywords:** Sedation, mechanical ventilation, sepsis, septic shock, dexmedetomidine, meta-analysis

## Abstract

**Purpose:**

Dexmedetomidine (DEX) is a central sympatholytic with sedative properties widely used in critically ill patients. However, its effects in patients with sepsis and septic shock remain controversial. This meta-analysis evaluated the efficacy and safety of DEX compared to other sedatives in mechanically ventilated patients with sepsis and septic shock.

**Methods:**

A systematic search was conducted across PubMed, Embase, Scopus, and Cochrane Library from inception through May 1, 2025 for randomized controlled trials comparing DEX with other sedatives or placebo in mechanically ventilated patients with sepsis and septic shock. Primary outcomes included overall mortality and Sequential Organ Failure Assessment (SOFA) scores. Secondary outcomes encompassed duration of mechanical ventilation (MV), length of stay in Intensive Care Unit (ICU), incidence of hypotension and bradycardia.

**Results:**

Fifteen studies involving 3,882 patients (1,945 in the DEX group, 1,937 in the control group) were included. DEX was demonstrated no significant differences compared to other sedatives or placebo in overall mortality (Risk Ratio [RR] 0.98, 95% Confidence Interval [CI] 0.90 to 1.07, *p* = 0.71, I^2^ = 0%), SOFA scores (Mean Difference [MD] − 0.14, 95% CI −0.81 to 0.52, *p* = 0.67, I^2^ = 0%), length of stay in ICU (MD −0.32, 95% CI −1.69 to 1.06, *p* = 0.65, I^2^ = 77%), or incidence of hypotension (RR 1.15, 95% CI 0.81 to 1.62, *p* = 0.44, I^2^ = 14%). However, DEX significantly reduced the duration of MV (MD −0.54, 95% CI −0.98 to −0.10, *p* = 0.02, I^2^ = 25%) but was associated with an increased incidence of bradycardia (RR 1.67, 95% CI 1.22 to 2.28, *p* = 0.001, I^2^ = 0%).

**Conclusions:**

In mechanically ventilated patients with sepsis and septic shock, DEX shortened duration of MV but was associated increased bradycardia risk. No mortality or organ dysfunction benefits were observed. These findings suggest DEX is a reasonable therapeutic option to facilitate earlier ventilator weaning in selected patients (particularly those without shock), but careful monitoring for cardiovascular adverse effects is warranted.

## Introduction

Sepsis, defined as life-threatening organ dysfunction caused by dysregulated host response to infection, remains a leading cause of mortality in critically ill patients [1,2], particularly those requiring mechanical ventilation (MV) [3]. In this population, appropriate sedation is used to ensure patient comfort and to attenuate physiological stress responses [4,5]. Traditionally, γ-aminobutyric acid (GABA) receptor agonists, including propofol and midazolam, have been the mainstay sedative agents in intensive care unit (ICU) settings [6–8]. Dexmedetomidine (DEX), a highly selective α_2_-adrenergic receptor agonist, has emerged as an alternative sympatholytic agent with distinct pharmacological properties, including analgesic-sparing effects, preservation of respiratory drive mimicking non-rapid eye movement (NREM) sleep, and potential anti-inflammatory benefits [9]. Despite these characteristics, its impact on clinically meaningful outcomes in patients with sepsis and septic shock remains uncertain, with conflicting evidence regarding effects on mortality, organ dysfunction, and safety [10,11].

Previous meta-analyses have reported inconsistent findings regarding DEX’s efficacy in septic patients. While one analysis of 19 randomized controlled trials (RCTs) suggested DEX might reduce mortality through modulation of inflammatory pathways [12], others found no significant survival benefit compared to conventional sedatives [13–15]. Similarly, its impact on Sequential Organ Failure Assessment (SOFA) scores has been equivocal, with heterogeneity across studies attributed to variations in dosing regimens, patient populations, and disease severity [15,16]. Some trials have reported shortened MV duration with DEX use, potentially due to maintain patients in a rousable state that facilitates communication and assessment of mental status [13,14]. However, as a primary sympatholytic agent, DEX requires careful assessment of volume status and cardiac conduction prior to administration [12].

Recently, several RCTs have investigated the effect of DEX on inflammatory mediators and organ dysfunction in critically ill patients with sepsis and septic shock [17–22]. Despite these investigations, critical knowledge gaps remain. First, prior meta-analyses often included studies with heterogeneous methodologies or limited sample sizes, constraining the robustness of conclusions. Second, existing evidence predominantly focuses on short-term outcomes, with limited data on the organ dysfunction endpoint. Third, the differential effects of DEX across the sepsis severity spectrum (sepsis versus septic shock) have not been systematically evaluated. Therefore, this meta-analysis aimed to synthesize current evidence from RCTs to evaluate the efficacy and safety of DEX versus alternative sedatives in mechanically ventilated patients with sepsis and septic shock.

## Methods

### Study design and registration

This meta-analysis was conducted according to the Preferred Reporting Items for Systematic Reviews and Meta-Analyses (PRISMA) 2020 guidelines [23] (Supplementary Material 1). The protocol was prospectively registered on the Open Science Framework (https://osf.io/2txpg).

### Search strategy

We performed a comprehensive systematic literature search of PubMed, Embase, Scopus, and Cochrane Library databases from inception through May 1, 2025. The search strategy combined medical subject headings and free-text terms related to “dexmedetomidine”, “sepsis”, “mechanical ventilation”, and “randomized controlled trial”. Complete search strategies for all databases are provided in Supplementary Material 2.

### Eligibility criteria

Inclusion criteria: (1) Population: adult patients (≥18 years) with sepsis and/or septic shock receiving invasive mechanical ventilation (MV) and intravenous sedation. Sepsis and septic shock are defined according to the Sepsis 3.0 [1] or Sepsis 1.0 [24], where sepsis is life-threatening organ dysfunction caused by a dysregulated host response to infection, and septic shock is a subset of sepsis in which underlying circulatory and cellular/metabolic abnormalities are profound enough to substantially increase mortality; (2) Intervention: intravenous DEX administration regardless of dose, initiation timing, or duration; (3) Comparison: other intravenous sedative drugs or placebo regardless of dose, initiation timing, or duration; (4) Outcomes: primary outcomes included overall mortality (ICU, hospital, 28/30-day, or 90-day mortality, when a trial reported mortality at multiple follow-up periods, we used the longest follow-up period for overall mortality assessment) and post-intervention SOFA scores (measured closest to Days 1 to 7 after randomization); secondary outcomes included MV duration, ICU length of stay, and incidence of severe adverse events (hypotension and bradycardia); (5) Study design: RCTs.

Exclusion criteria: non-randomized studies, pediatric populations, studies with incomplete outcome data, patients receiving only non-invasive ventilation or high-flow nasal oxygen, and studies not involving mechanically ventilated patients receiving intravenous sedation.

### Data extraction and quality assessment

Two independent reviewers extracted data using standardized forms, including study characteristics (authors, publication year, sample size), population demographics, intervention details (DEX dosage, comparator agents), sedation targets, and outcome measures. To ensure population specificity, for RCTs enrolling broad, mixed ICU populations (e.g. general medical or surgical ICU cohorts), we strictly extracted outcomes only for the specific subgroup of patients with a confirmed diagnosis of sepsis. Data for these subgroups were obtained from the primary analysis stratified by diagnosis or from supplementary appendices provided by the original authors. The identification of sepsis cases in these mixed trials was based on the definitions adhered to by the original study protocols (e.g. Sepsis-3 or Sepsis-1 criteria), whether adjudicated primarily or *via* prespecified subgroup analysis. Furthermore, we verified that all included patients, including those in extracted subgroups, were receiving mechanical ventilation at the time of randomization. When data were presented as median with interquartile range (IQR), values were transformed to mean and standard deviation using the established methodology proposed by Wan et al. [25].

Methodological quality was assessed using the Cochrane Risk of Bias Tool (RoB 2.0) across five domains: randomization process, deviations from intended interventions, missing outcome data, measurement of outcomes, and selection of reported results [26]. The certainty of the evidence was classified using the Grading of Recommendations Assessment, Development and Evaluation (GRADE) approach, using GRADEpro GDT software [27]. Disagreements in data extraction and quality assessment were resolved through consensus discussion with a third adjudicator.

### Statistical analysis

Statistical analyses were performed using Review Manager (version 5.3) and R software (version 4.3.1) with the “meta” and “robvis” package [28,29]. Dichotomous outcomes (mortality, hypotension, bradycardia) were analyzed using risk ratios (RRs) with 95% confidence intervals (CIs). Continuous outcomes (SOFA score, duration of MV, length of stay in ICU) were evaluated using mean differences (MDs) with 95% CI. Prior to pooling, all time-dependent variables (e.g. duration of MV, length of stay) were harmonized to “days”; values reported in hours were divided by 24. Given expected clinical heterogeneity among trials, we employed the DerSimonian-Laird random-effects model for all analyses. Between-study heterogeneity was assessed using inconsistency (I^2^) statistics [30], with I^2^ value > 30% indicating substantial heterogeneity. Publication bias was evaluated using funnel plots and Egger’s regression test (restricted to outcomes with ≥ 10 studies), with trim-and-fill methodology employed when bias was detected.

Prespecified subgroup analyses (defined prospectively in the protocol) were conducted by population (sepsis versus septic shock), and comparator agent (propofol, other sedatives, or placebo) to explore heterogeneity sources. We acknowledge that performing multiple subgroup comparisons increases the potential risk of type I error. No formal statistical adjustments for multiplicity (e.g. Bonferroni correction) were applied. Therefore, findings from these secondary analyses should be interpreted as exploratory and hypothesis-generating. Sensitivity analyses examined individual study influence through sequential exclusion methods. Given the potential for skewed data in time-to-event outcomes, we performed analyses excluding trials where means and standard deviations were estimated from medians and interquartile ranges, as well as checking consistency using log-transformed means where data allowed. Second, to address potential underestimation of between-study variance in small sample sizes, we repeated the meta-analysis using the Restricted Maximum Likelihood (REML) estimator alongside the standard DerSimonian-Laird method. Finally, we conducted sensitivity analyses excluding studies classified as “high risk of bias” and those with significant deviations from intended interventions.

## Results

### Study selection and characteristics

**Figure 1.**
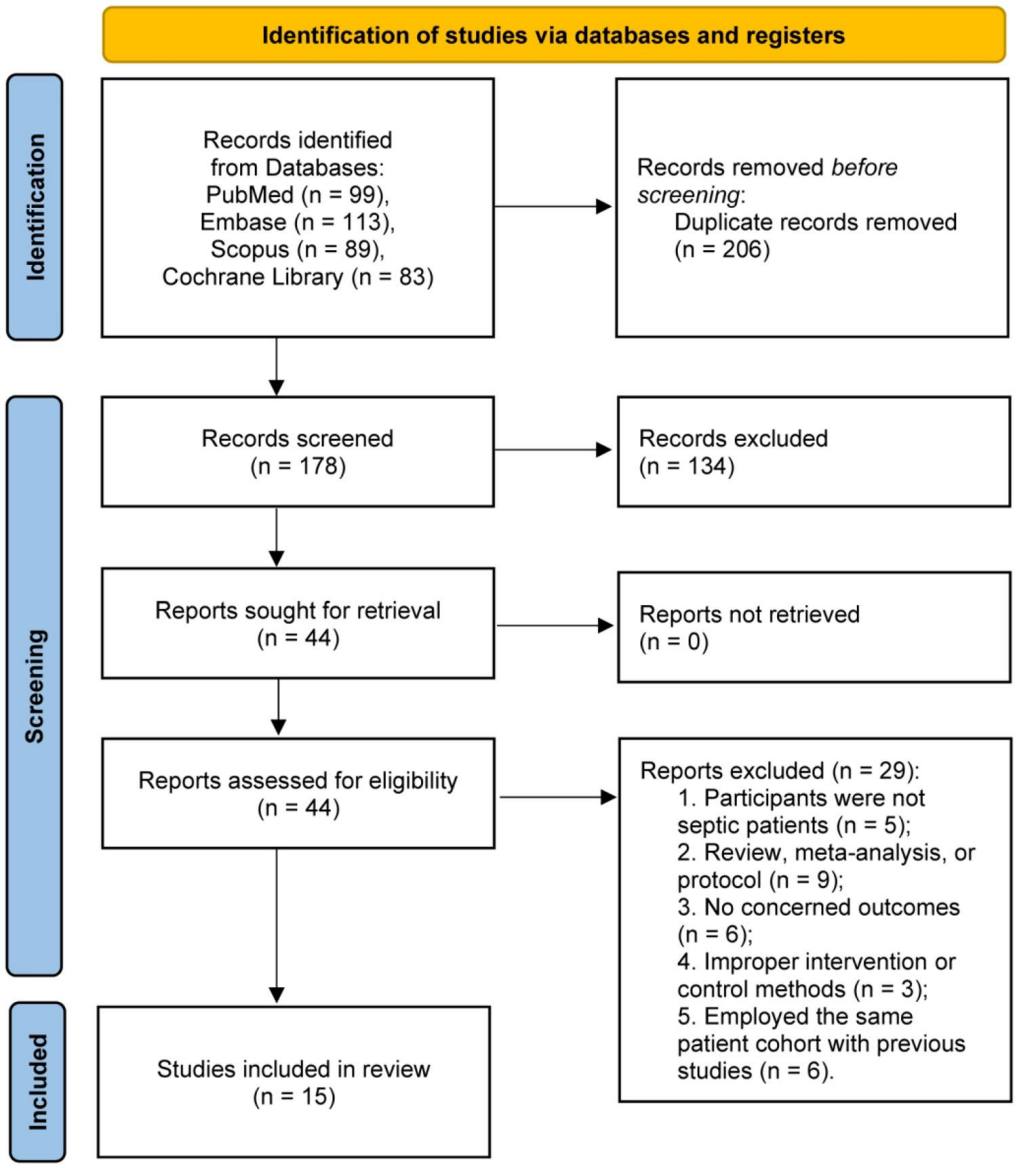
PRISMA 2020 flow diagram for this meta-analysis.

The systematic search identified 384 records from electronic databases. After duplicate removal, 178 records underwent title and abstract screening. Following full-text assessment of 44 articles, 15 RCTs [17–22,31–39] met inclusion criteria and were included in the meta-analysis (Figure 1).

Study characteristics are summarized in Table 1. For large mixed-population trials such as Maximizing Efficacy of Targeted Sedation and Reducing Neurological Dysfunction (MENDS) [34], and Sedation Practice in Intensive Care Evaluation (SPICE III) [36], data were extracted exclusively from the defined sepsis strata. These trials collectively enrolled 3,882 patients, with 1,945 patients assigned to the DEX group and 1,937 patients assigned to the control group. The publication years ranging from 2007 to 2025 and sample sizes varying from 24 to 2,495 patients. Six studies [19,21,31,33,34,39] exclusively enrolled patients with sepsis, seven studies specifically targeted septic shock populations [17,18,20,22,32,37,38], and two [35,36] enrolled mixed cohorts. The included trials compared DEX with various control drugs, including propofol (*n* = 8 RCTs) [19,21,32,33,35,36,38,39], placebo (*n* = 2 RCTs) [20,37], midazolam or other sedative agents (*n* = 5) RCTs [17,18,22,31,34]. The detailed intervention protocols, sedation targets, allowance of background sedatives or rescue boluses, and baseline vasopressor exposure are summarized in Supplementary Material 3, indicating substantial between‑trial variability in sedation depth, drug dosing, and co‑interventions.

**Table 1.** Characteristics of included studies.

Study, year	Design	Patients	Intervention and control methods	Sedation goals
Mokhlesian 2025	Single-center, double-blind	48 patients with septic shock (Sepsis 3.0)	Intervention: DEX at 0.2 to 2.5 μg/kg·h, for 24 h; Control: morphine or midazolam at 0.5 to 5 mg/h	RASS score of −2 to 0
ADRESS trial 2025	Multicenter, double-blind	32 patients with septic shock (Sepsis 3.0)	Intervention: DEX at 1 μg/kg·h, for 24 h; Control: placebo at same rate (5% glucose)	NR
Iten 2025	Single-center, open-label	67 patients with sepsis (Sepsis 3.0)	Intervention: DEX at 0.1 to 1.4 μg/kg·h, for 7 days; Control: propofol and midazolam determined by treating clinician	Determined by treating clinician
Patidar 2024	Single-center, double-blind	54 patients with sepsis (Sepsis 3.0)	Intervention: DEX at 0.15 to 1.5 μg/kg·h, for 7 days; Control: propofol at 5 to 50 μg/kg·h	RASS score of −2 to 0
Ezz Al-Regal 2024	Single-center, open-label	90 patients with septic shock (Sepsis 3.0)	Intervention: DEX at 0.2 to 0.7 μg/kg·h, for 2 days; Control: conventional sedation	RASS score of −2 to 0
Elayashy 2023	Single-center, double-blind	24 patients with septic shock (Sepsis 3.0)	Intervention: DEX at 0.15 to 0.75 μg/kg·h, for 24 h; Control: midazolam at 1 to 5 mg/h	RASS score of −3 to −1
MENDS2 trial 2021	Multicenter, double-blind	422 patients with sepsis (Sepsis 3.0)	Intervention: DEX at 0.15 to 1.5 μg/kg·h, for 14 days; Control: propofol at 5 to 50 μg/kg·h	RASS score of −2 to 0
Gheibi 2020	Single-center, single-blind	66 patients with septic shock (Sepsis 3.0)	Intervention: DEX at 0.6 μg/kg·h, for 12 h; Control: placebo at same rate (normal saline)	NR
Liu 2020	Single-center, open-label	200 patients with septic shock (Sepsis 3.0)	Intervention: DEX at 0.2 to 0.3 μg/kg·h, for 5 days; Control: propofol at 1 to 3 mg/kg·h	RASS score of −2 to 0
SPICE III trial 2019	Multicenter, open-label	2495 patients with sepsis (Sepsis 3.0)	Intervention: DEX at 0.1 to 1.5 μg/kg·h, for 28 days; Control: propofol directed by the treating physician	RASS score of −2 to +1
DESIRE trial 2017	Multicenter, open-label	201 patients with sepsis (Sepsis 1.0)	Intervention group: DEX at 0.1 to 0.7 μg/kg·h, for 28 days; Control group: propofol at 0 to 3 mg/kg/h or midazolam at 0 to 0.15 mg/kg/h	RASS score of −2 to 0
MENDS trial 2010	Multicenter, double-blind	63 patients with sepsis (Sepsis 1.0)	Intervention group: DEX at 0.1 to 1.5 μg/kg·h, for 5 days; Control group: lorazepam at 0 to 10 mg/h	RASS score of −2 to +1
Tasdogan 2009	Single-center, open-label	40 patients with sepsis (Sepsis 1.0)	Intervention group: DEX at 0.2–2.5 μg/kg·h, for 24 h; Control group: propofol at 1 to 3 mg/kg·h	RSS ≤ 2
Memis 2009	Single-center, open-label	40 patients with septic shock (Sepsis 1.0)	Intervention group: DEX at 0.2 to 2.5 μg/kg·h, for 24 h; Control group: propofol at 1 to 3 mg/kg·h	RSS ≤ 2
Memis 2007	Single-center, open-label	40 patients with sepsis (Sepsis 1.0)	Intervention group: DEX at 0.2–2.5 μg/kg·h, for 24 h; Control group: midazolam at 0.1–0.5 mg/kg·h	RSS < 2

Abbreviation: DEX: Dexmedetomidine; RASS: Richmond agitation sedation scale; RSS: Ramsay sedation score; NR: Not reported.

Note: For mixed-population trials (e.g. SPICE III, MENDS), only data from the sepsis subgroup were extracted and analyzed. Sepsis diagnosis criteria (e.g. Sepsis-1.0, Sepsis-3.0) denote the definition used for study inclusion or subgroup classification in the original trial.

### Risk of bias assessment

**Figure 2.**
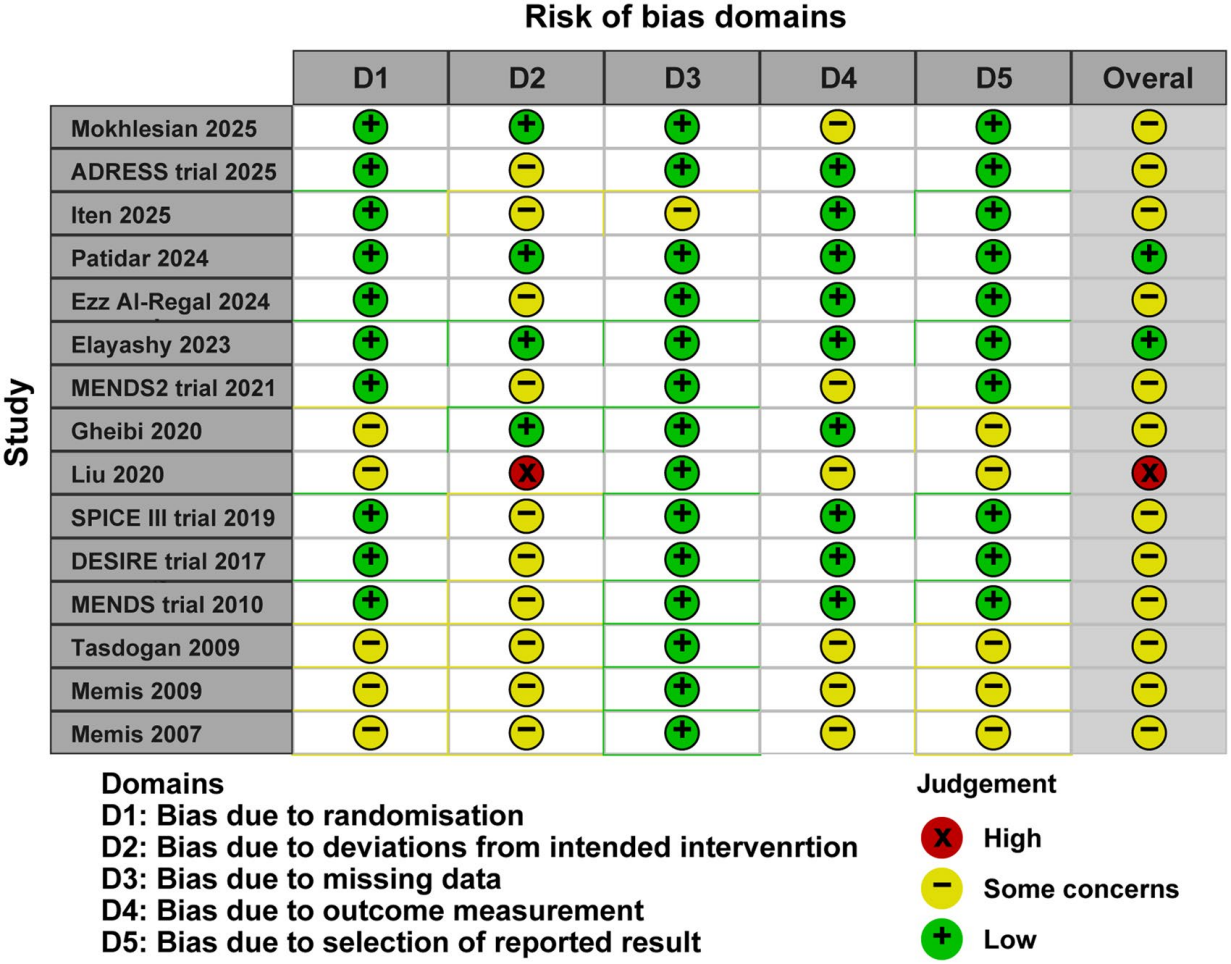
Risk of bias 2 of all included studies.

Using RoB 2.0 criteria, two studies were classified as low risk of bias, 12 as having some concerns, and one as high risk (Figure 2). Most studies demonstrated low bias risk in randomization processes, missing outcome data, outcome measurement, and selective reporting domains. The domain most frequently triggering a classification of “some concerns” was Domain 2 (deviations from the intended interventions). This was largely attributable to the open-label design inherent in many critical care sedation trials, where blinding of the titrated intervention was not feasible. To assess the impact of these methodological concerns on our certainty, we conducted sensitivity analyses, which demonstrated that excluding the study classified as high risk or those with specific protocol deviations did not materially alter the primary findings.

### Overall mortality

**Figure 3.**
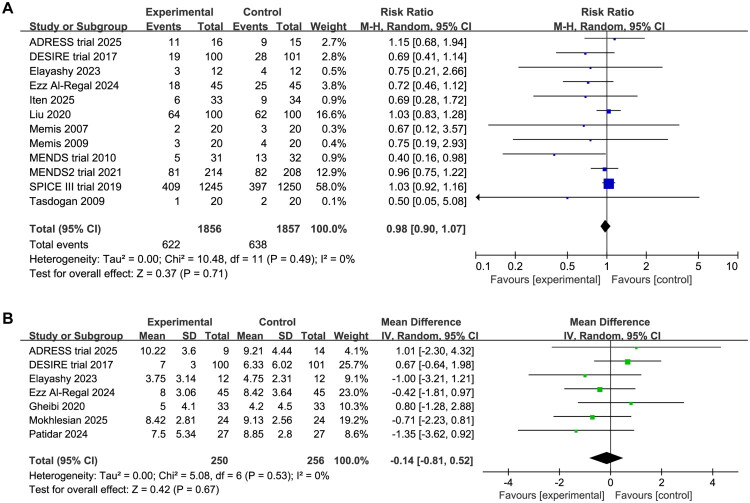
Forest plot comparing the effect of DEX versus comparative drugs on (A) overall mortality, (B) SOFA score.

Twelve trials with a total of 3,713 patients reported mortality data (1,856 in DEX group, 1,857 in control group). The mortality rates were 33.5% (622/1856) in the DEX group and 34.4% (638/1857) in the control group. There was no statistically significant difference in mortality between patients receiving DEX and those receiving other sedative agents (RR 0.98, 95%CI 0.90 to 1.07, *p* = 0.71, I^2^ = 0%, Figure 3(A)). Publication bias assessment revealed potential bias for overall mortality, and trim-and-fill analysis produced symmetrical funnel plots and confirmed no association between DEX and overall mortality (RR 1.02, 95%CI 0.94 to 1.11, *p* = 0.20, I2 = 21%, Supplementary Material 4). The certainty of evidence was low due to methodological weaknesses and publication bias in studies (Table 2). Consistent nonsignificant effects were observed across all prespecified mortality endpoints: ICU mortality (RR 0.98, 95%CI 0.90 to 1.07, *p* = 0.71, I^2^ = 0%), 28/30-day mortality (RR 0.98, 95%CI 0.90 to 1.07, *p* = 0.71, I^2^ = 0%), 90-day mortality (RR 0.98, 95%CI 0.90 to 1.07, *p* = 0.71, I^2^ = 0%, Supplementary Material 5).

**Table 2.** Certainty of the evidence according to GRADE.

Outcomes	No. of participant (studies)	Certainty of the evidence (GRADE)	Relative effect (95% CI)	Anticipated absolute effects	
				Risk with other sedatives or placebo	Risk difference with Dexmedetomidine
Overall mortality	3713 (12 RCTs)	⨁⨁◯◯ Low^a,b^	**RR 0.98** (0.90 to 1.07)	344 per 1,000	**7 fewer per 1,000** (34 fewer to 24 more)
SOFA score	506 (7 RCTs)	⨁⨁⨁◯ Moderate^a^	–		MD **0.14 points lower** (0.81 lower to 0.52 higher)
Duration of mechanical ventilation	1181 (9 RCTs)	⨁⨁⨁◯ Moderate^a^	–		MD **0.54 days lower** (0.98 lower to 0.1 lower)
Length of ICU stay	1329 (11 RCTs)	⨁◯◯◯ Very low^a,b,c^	–		MD **0.32 days lower** (1.69 lower to 1.06 higher)
Incidence of bradycardia	925 (8 RCTs)	⨁⨁⨁◯ Moderate^a^	**RR 1.67** (1.22 to 2.28)	107 per 1,000	**72 more per 1,000** (24 more to 137 more)
Incidence of hypotension	631 (5 RCTs)	⨁⨁◯◯ Low^a, d^	**RR 1.15** (0.81 to 1.62)	413 per 1,000	**62 more per 1,000** (78 fewer to 256 more)
*The risk in the intervention group (and its 95% confidence interval) is based on the assumed risk in the comparison group and the relative effect of the intervention (and its 95% CI). CI: confidence interval; MD: mean difference; RR: risk ratio.					
GRADE Working Group grades of evidence High certainty: we are very confident that the true effect lies close to that of the estimate of the effect. Moderate certainty: we are moderately confident in the effect estimate: the true effect is likely to be close to the estimate of the effect, but there is a possibility that it is substantially different. Low certainty: our confidence in the effect estimate is limited: the true effect may be substantially different from the estimate of the effect. Very low certainty: we have very little confidence in the effect estimate: the true effect is likely to be substantially different from the estimate of effect.					

Explanations. Studies without blinded therapists or assessment. Publication bias. High heterogeneity. High imprecision.

The bolded text is used solely to enhance the prominence of the results, with no special significance attached.

Subgroup analyses (Supplementary Material 6) demonstrated no statistically significant effect on overall mortality between DEX and control groups in patients with sepsis (RR 0.99, 95%CI 0.88 to 1.12, *p* = 0.88, I^2^ = 3%) or septic shock (RR 0.93, 95%CI 0.79 to 1.10, *p* = 0.40, I^2^ = 0%). Compared with propofol, DEX showed no difference on overall mortality (RR 1.00, 95%CI 0.92 to 1.10, *p* = 0.94, I^2^ = 0%). However, DEX was associated with reduced overall mortality when compared to other sedatives collectively (RR 0.65, 95%CI 0.45 to 0.94, *p* = 0.02, I^2^ = 0%).

### SOFA score after intervention

Data on SOFA scores were available from seven trials including 506 patients (250 in DEX group, 256 in control group). The timing of SOFA score measurements varied across trials: on day 3 (*n* = 5), day 5 (*n* = 1), and day 7 (*n* = 1) after the intervention. The pooled analysis revealed no statistically significant difference in SOFA scores between the DEX and control groups (MD −0.14, 95%CI −0.81 to 0.52, *p* = 0.67, I^2^ = 0%, Figure 3(B)). No evidence of publication bias was observed, as assessed by visual inspection of the funnel plot. The evidence for SOFA scores was graded as moderate certainty, primarily downgraded due to methodological weaknesses (Table 2). Moreover, there was no significant imbalances for baseline SOFA scores between the intervention and control arms at randomization.

Prespecified subgroup analyses (Supplementary Material 6) indicated no SOFA score benefit with DEX in patients with sepsis (MD −0.12, 95%CI −2.05 to 1.81, *p* = 0.90, I^2^ = 56%) or septic shock (MD −0.31, 95%CI −1.13 to 0.52, *p* = 0.47, I^2^ = 0%). Direct comparator-based subgroups further showed DEX had no significant SOFA improvement versus propofol (MD −0.12, 95%CI −2.05 to 1.81, *p* = 0.90, I^2^ = 56%) or other sedatives (MD −0.62, 95%CI −1.56 to 0.30, *p* = 0.18, I^2^ = 0%).

### Duration of MV and length of ICU stay

**Figure 4.**
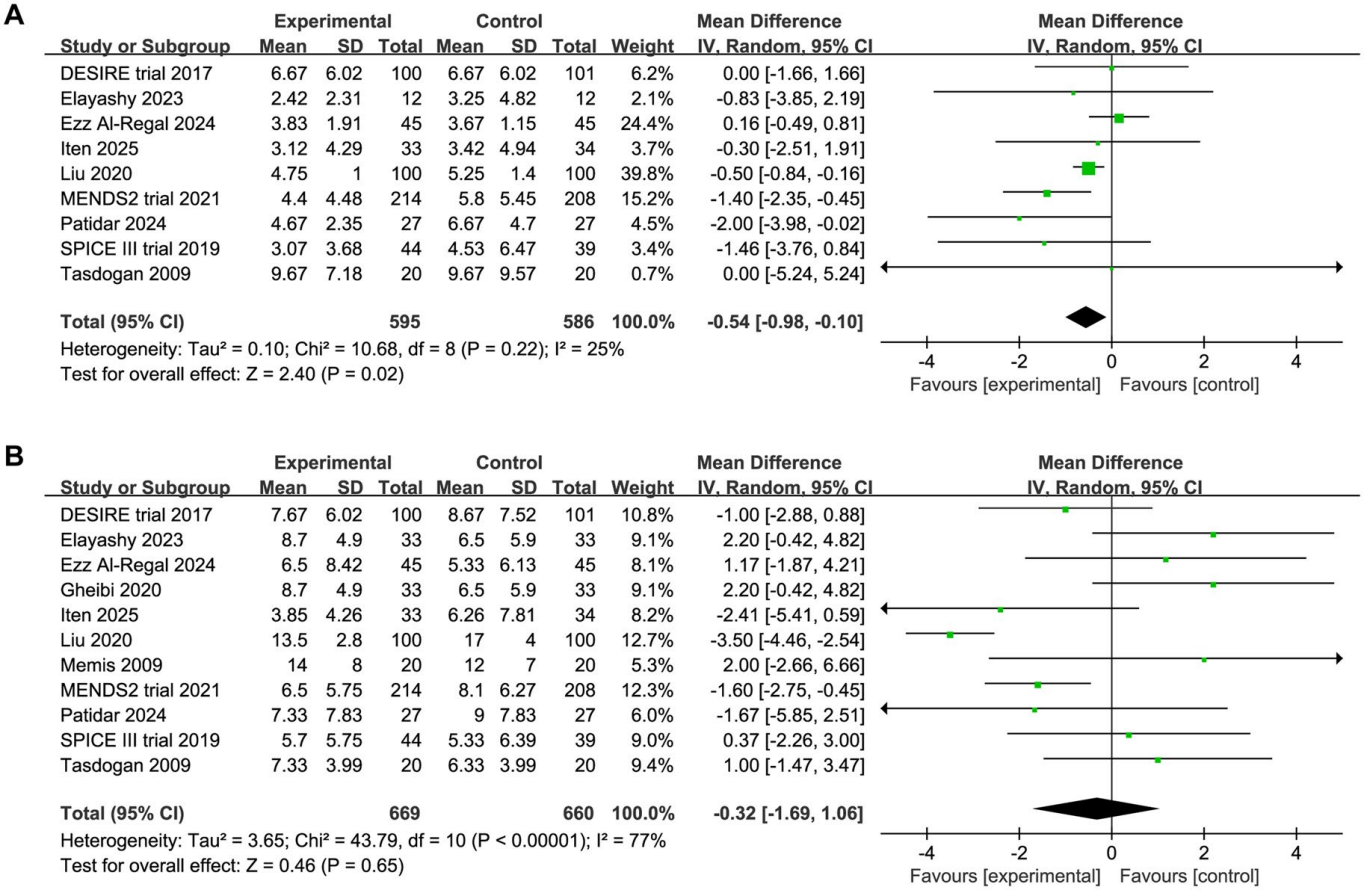
Forest plot comparing the effect of DEX versus comparative drugs on (A) duration of MV, (B) length of ICU stay.

Nine studies with 1,181 patients (595 in DEX group, 586 in control group) provided data on mechanical ventilation duration. Patients receiving DEX had significantly shorter mechanical ventilation duration compared to controls (MD −0.54, 95%CI −0.98 to −0.10, *p* = 0.02, I^2^ = 25%, Figure 4(A)). Data on length of ICU stay were reported in 11 studies encompassing 1329 patients (669 in DEX group, 660 in control group). No significant difference was observed between the DEX and control groups (MD −0.32, 95%CI −1.69 to 1.06, *p* = 0.65, I^2^ = 77%, Figure 4(B)). Publication bias assessment revealed potential bias for length of ICU stay, and trim-and-fill analysis produced symmetrical funnel plots and showed reduced ICU length of stay (MD −2.05, 95%CI −3.68 to −0.42, *p* < 0.01, I^2^ = 83%, Supplementary Material 4). Using the GRADE framework (Table 2), we found moderate certainty evidence that DEX reduces the duration of MV compared to controls. However, the evidence for length of ICU stay was graded as very low, primarily due to methodological weaknesses, publication bias and high heterogeneity.

Prespecified subgroup analyses (Supplementary Material 6) revealed significant heterogeneity in the efficacy of DEX based on sepsis severity and comparator sedatives. In septic patients without shock, DEX significantly reduced the duration of MV (MD −1.08, 95%CI −1.79 to −0.36, *p* = 0.003, I^2^ = 0%) and length of ICU stay (MD −1.21, 95%CI −2.12 to −0.31, *p* = 0.009, I^2^ = 5%). In contrast, no benefit was observed in septic shock patients for duration of MV (MD −0.33, 95%CI −0.80 to 0.14, *p* = 0.17, I^2^ = 26%) or length of ICU stay (MD 0.57, 95%CI −2.16 to 3.29, *p* = 0.68, I^2^ = 87%). Compared with propofol, DEX significantly reduced the duration of MV (MD −0.64, 95%CI −0.97 to −0.31, *p* = 0.0001, I^2^ = 2%), no significant difference was observed when DEX was compared to non-propofol sedatives.

### Bradycardia and hypotension

**Figure 5.**
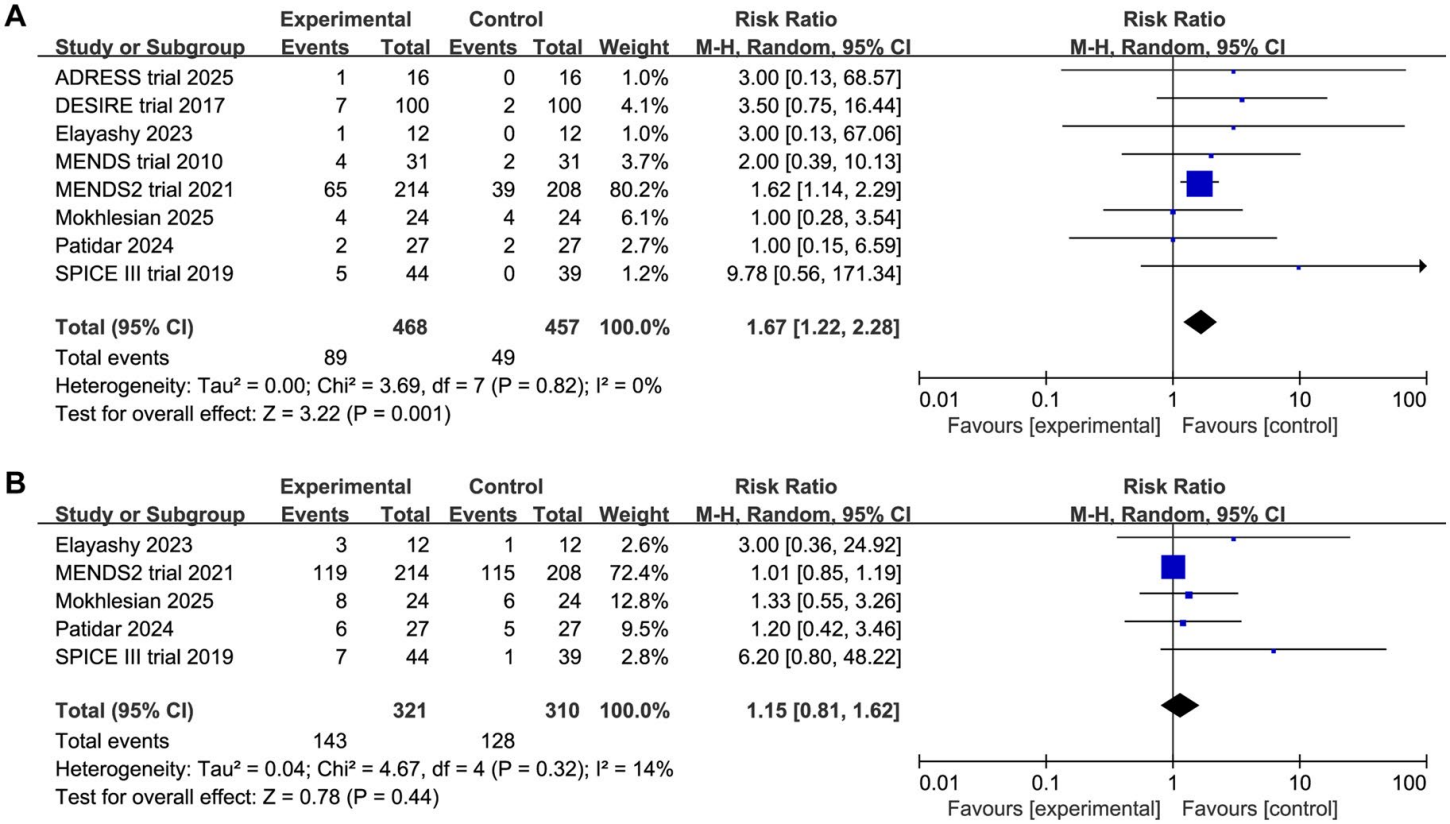
Forest plot comparing the effect of DEX versus comparative drugs on (A) bradycardia, (B) hypotension.

Eight studies patients reported bradycardia and hypotension as adverse events. Consistent with the sympatholytic mechanism of DEX, the analysis revealed a significantly higher risk of bradycardia (RR 1.67, 95%CI 1.22 to 2.28, *p* = 0.001, I^2^ = 0%, Figure 5(A)). However, no significant increase in the incidence of hypotension was observed (RR 1.15, 95% CI 0.81 to 1.62, *p* = 0.44, I^2^ = 14%, Figure 5(B)). Publication bias assessment revealed potential bias for incidence of bradycardia and hypotension, and trim-and-fill analysis produced symmetrical funnel plots were consistent with the original analysis (bradycardia: RR 1.57, 95%CI 1.16 to 2.13, *p* = 0.003, I^2^ = 0%; hypotension: RR 1.01, 95%CI 0.86 to 1.19, *p* = 0.26, I^2^ = 21%, Supplementary Material 4). The certainty of evidence was moderate for bradycardia and low for hypotension to methodological weaknesses and high imprecision in studies (Table 2).

Subgroup analyses (Supplementary Material 6) indicated significant heterogeneity in bradycardia based on sepsis severity and comparator sedatives. In septic patients without shock, DEX significantly increased the incidence of bradycardia (RR 1.67, 95%CI 1.20 to 2.31, *p* = 0.002, I^2^ = 0%), whereas no increased risk was observed in septic shock patients (RR 1.70, 95%CI 0.61 to 4.73, *p* = 0.31, I^2^ = 0%). Sedative-specific comparisons further demonstrated that the elevated bradycardia risk was primarily driven by direct comparisons with propofol (RR 1.69, 95%CI 1.22 to 2.36, *p* = 0.002, I^2^ = 0%), but not evident against non-propofol sedatives.

### Post‑hoc subgroup analysis by duration of intervention

We conducted a post‑hoc subgroup analysis stratifying trials according to the duration of DEX administration. Studies were categorized as short‑duration exposure (<3 days) or prolonged exposure (≥3 days), based on the reported planned duration of the intervention (Supplementary Material 6). Prolonged administration was associated with a significant reduction in the duration of MV (MD −0.75, 95%CI −1.23 to −0.27, *p* = 0.002, I^2^ = 17%). A concomitant reduction in length of ICU stay was also observed in this subgroup (MD −1.83, 95%CI −3.07 to −0.58, *p* = 0.004, I^2^ = 63%). In contrast, trials in which DEX was administered for <3 days showed no reduction in MV duration and were associated with a longer ICU length of stay compared with controls (MD 1.68, 95%CI 0.40 to 2.96, *p* = 0.01, I^2^ = 0%). With respect to safety outcomes, prolonged DEX exposure was associated with a higher incidence of bradycardia (RR 1.71, 95%CI 1.23 to 2.36, *p* = 0.001, I^2^ = 0%), whereas no significant difference in bradycardia or hypotension was observed in the short‑duration subgroup. Furthermore, no meaningful interaction was observed between duration‑based subgroups and overall mortality or SOFA scores, and all duration‑based findings should be interpreted in light of their post‑hoc nature and limited number of contributing trials.

### Sensitivity analyses

Sensitivity analyses confirmed the robustness of overall mortality, SOFA score, length of ICU stay and incidence of hypotension, as sequential exclusion of individual studies did not materially alter the effect size or statistical significance (Supplementary Material 7). However, the originally observed reduction in duration of MV lost statistical significance after sequential removal of four RCTs [19,36, 38,39], the increased risk of bradycardia became non-significant following exclusion of one RCT [39]. Moreover, the application of the REML estimator yielded results consistent with the primary DerSimonian–Laird analysis across all concerned outcomes, suggesting that the variance estimation method did not bias the conclusions (Supplementary Material 7). Furthermore, sensitivity analyses addressing skewed data distributions, specifically extracting studies requiring median-to-mean conversion for SOFA score, duration of MV, and length of ICU stay, did not materially alter the effect sizes. Similarly, exclusion of the high-risk-of-bias study intensified the confidence in the null results for outcomes (Supplementary Material 7).

## Discussion

In this meta-analysis of 15 RCTs involving 3,882 mechanically ventilated patients with sepsis or septic shock (1,945 in DEX group, 1,937 in control group), DEX was associated with a modest reduction in the duration of MV but an increased risk of bradycardia. In contrast, no clear benefits were observed for overall mortality, post-intervention SOFA score, length of ICU stay, or hypotension. Overall, these findings suggest that DEX may facilitate ventilator liberation in selected patients, but it does not consistently improve survival or organ dysfunction outcomes at the trial level and therefore requires careful cardiovascular monitoring.

### Clinical implications of key findings

The observed reduction in duration of MV is consistent with the landmark RCT by Ruokonen et al. [40], which reported shorter duration of MV with DEX during long-term sedation in mechanically ventilated ICU patients. From a mechanistic perspective, the ventilatory and weaning advantages of DEX are supported by early physiological work on central α_2_-adrenergic agonism. Ghignone et al. first demonstrated in humans that perioperative α2-agonist administration was associated with improved hemodynamic stability together with reduced anesthetic/opioid requirements [41]. Subsequently, Flacke et al. reported related findings in cardiac surgery, showing reduced narcotic requirement and improved hemodynamic/adrenergic stability with clonidine [42]. Taken together with experimental data showing that dexmedetomidine and clonidine can sustain activation of respiratory rhythm generation [43], these observations provide a coherent physiological rationale for the shorter duration of mechanical ventilation observed in prior trials and in the present meta-analysis. This aligns with previous findings in general ICU populations and reinforces the potential of DEX to support timely extubation [44,45]. Despite this benefit, DEX did not improve short-term mortality or organ dysfunction, as measured by SOFA scores. This may reflect the complex, multifactorial nature of sepsis pathophysiology, in which sedation strategy is only one component of patient management. Furthermore, the duration of sedative exposure is relatively short compared to the prolonged inflammatory and immunological disturbances in sepsis, potentially limiting its impact on survival outcomes.

Our subgroup analyses did not demonstrate a statistically significant reduction in mortality or organ dysfunction among patients with septic shock. However, these findings should not be interpreted as evidence against the potential clinical benefit of DEX in more severely ill patients. Prior randomized trials including the study by Kawazoe et al. [35] suggest that α2-agonists may exert beneficial effects in selected patients with severe sepsis or septic shock, particularly when integrated into a broader hemodynamic and ventilatory management strategy. The apparent discrepancy between these observations and our pooled estimates may be explained by several factors. First, subgroup analyses in the present meta-analysis were exploratory and underpowered, with limited sample sizes and substantial clinical heterogeneity. Second, differences in illness severity, timing of DEX initiation, co-administration of other sedatives, and hemodynamic optimization strategies may critically influence treatment response. Finally, DEX is often used as part of a multimodal sedation and cardiovascular management approach rather than as an isolated intervention, which may dilute its detectable effect in trial-level analyses. Therefore, rather than contradicting prior evidence, our findings underscore the complexity of identifying patient subgroups most likely to benefit from DEX. Future studies specifically designed to evaluate DEX-based sedation strategies in well-characterized cohorts of patients with severe sepsis or septic shock are warranted. Regarding the comparison with specific agents, a significant reduction in mortality was observed specifically when DEX was compared against other sedative agents (RR 0.65, 95%CI 0.45 to 0.94, *p* = 0.02). From a clinical perspective, this indicates that the survival benefit of DEX is most evident when it replaces other continuous infusions (such as midazolam or other sedative agents). This finding supports the concept that avoiding the accumulation and deep sedation associated with traditional agents contributes to better outcomes, rather than DEX simply being superior to standard care where sedation might be minimal.

DEX exerts sympatholytic effects that manifest clinically as heart rate control. However, its relevance in critically ill patients should not be restricted to isolated cardiac effects. Rather, α2-agonist–based heart rate control must be interpreted within the context of overall systemic and microcirculatory physiology [46]. When administered using a cautious “start low, go slow” approach without bolus dosing [47], and combined with iterative echocardiographic assessment, careful volume optimization, and close monitoring for atrioventricular conduction disturbances, DEX may contribute to improved circulatory balance rather than isolated bradycardia.

Importantly, in septic patients without shock, endotracheal intubation and controlled MV should not be regarded as routine management strategies. Outside of impaired consciousness, the accepted indications for intubation remain established acute ventilatory failure or acute circulatory failure. Importantly, unnecessary intubation and positive pressure ventilation may themselves induce harm, particularly through adverse hemodynamic effects and increased circulatory stress [48]. Contemporary debates in critical care, including the Tobin et al. [49] vs. Gattinoni et al. [50] controversy during the coronavirus disease 2019 pandemic, have further underscored the importance of preserving spontaneous breathing whenever feasible. Within this framework, sedation strategies that facilitate tolerance of spontaneous breathing rather than promote controlled ventilation may be particularly relevant.

The post‑hoc analyses suggest that the clinical effects of DEX may be duration‑dependent. Trials employing prolonged administration demonstrated reductions in both mechanical ventilation duration and ICU length of stay, whereas shorter exposure conferred no benefit and was associated with longer ICU stays. These findings align with the concept that sustained α_2_‑agonist-mediated sympatholysis and sleep‑mimicking sedation may require sufficient cumulative exposure to translate into clinically meaningful effects. However, prolonged administration was also associated with an increased risk of bradycardia, underscoring the narrow therapeutic window of α_2_‑agonists in critically ill patients. This trade‑off highlights the importance of careful patient selection, gradual titration, and close hemodynamic monitoring when prolonged DEX sedation is considered.

### Comparison with previous literature

Our findings demonstrate substantial concordance with the most comprehensive meta-analysis to date examining DEX in critically ill patients requiring MV: Lewis et al. [45] analyzed 77 RCTs across diverse critically ill populations in ICUs. Their analysis revealed that DEX significantly reduced delirium incidence, shortened duration or MV and ICU length of stay, but increased the risk of bradycardia and hypotension [45]. Our results align closely with these findings, particularly regarding the reduction in duration or MV and increased risk of bradycardia, though we did not observe a significant decrease in length of ICU stay or increase in risk of hypotension in our sepsis-specific population. Our neutral findings regarding mortality are consistent with previous studies reporting no survival advantage with DEX, including the meta-analysis by Pasin et al. whose results closely resemble those of the present meta-analysis [51]. These discrepancies likely reflect differences in patient selection, illness severity, co-interventions, and hemodynamic management strategies, underscoring the context-dependent nature of α_2_-agonist–based sedation.

The overall mortality rates observed in our pooled results were 33.5% in the DEX group and 34.4% in the control group, which aligns with historical mortality data for sepsis and septic shock. This consistency strengthens the external validity of our findings. Previous studies have reported wide variability in mortality across cohorts with sepsis and septic shock. In particular, Lefrant et al. [52] reported a mortality of approximately 27% in a mixed sepsis/septic shock population, whereas higher rates (up to 70%) have been reported in cohorts with greater illness severity [53].

The existing literature has examined DEX’s effects in some specific patient populations, particularly in postoperative patients and cardiac surgery populations [54]. The evidence bases for DEX specifically in septic patients has remained relatively limited, with few dedicated meta-analyses and those that exist incorporating a limited number of RCTs [13–15,55]. Our investigation addresses this critical knowledge gap by providing the most current and comprehensive meta-analysis specifically examining DEX effects in septic patients requiring mechanical ventilation. Our subgroup analyses do not demonstrate a differential effect of DEX on mortality or SOFA scores between patients with sepsis and those with septic shock. Both subgroups showed neutral effects with overlapping confidence intervals. Therefore, our data do not support the notion that DEX is less effective or more harmful in septic shock, but rather indicate an absence of demonstrable benefit on these endpoints across the severity spectrum.

Furthermore, some included studies reported a large benefit of DEX, whereas others showed neutral effects. The most plausible explanation lies not in inconsistent pharmacology, but in differences in clinical practices and institutional policies. Trials reporting marked benefits typically implemented DEX as part of a coherent sedation strategy, characterized by early initiation, avoidance of deep sedation, limited use of concomitant sedatives, preservation of spontaneous breathing, and proactive hemodynamic optimization [34,35]. In contrast, in many multicenter or mixed-population studies, DEX was used as an adjunct to conventional sedation and applied across highly heterogeneous patient populations, thereby diluting any specific physiological signal.

This context dependency is further supported by observations in more homogeneous subgroups, such as acute respiratory distress syndrome [48], where standardized ventilatory and sedation practices allow the effects of α_2_-agonist-based sedation to become detectable. When diverse patient phenotypes and care strategies are pooled, as in broad ICU populations, the absence of a measurable effect should therefore be interpreted as a consequence of heterogeneity rather than evidence of inefficacy.

## Limitations

Several limitations warrant consideration in interpreting our results. An important limitation of the present meta‑analysis is the substantial heterogeneity in sedation practices across included trials. Studies differed markedly with respect to targeted sedation depth (ranging from light, protocolized Richmond Agitation-Sedation Scale targets to clinician‑determined sedation), DEX dosing strategies and duration, allowance of background sedatives or rescue boluses, and baseline exposure to vasopressors. These factors are particularly relevant for outcomes such as duration of MV and hemodynamic adverse events, and may introduce residual confounding at the trial level. Secondly, most studies were rated as having “some concerns,” mainly due to potential deviations from intended interventions in unblinded settings, introducing possible performance bias. Although sensitivity analyses supported the robustness of primary conclusions, this risk cannot be fully excluded. Thirdly, the duration‑based subgroup analysis was not prespecified and should therefore be regarded as exploratory. The cut‑off of 3 days, although clinically motivated, remains arbitrary, and residual confounding related to illness severity, co‑interventions, and sedation strategies cannot be excluded. Consequently, these findings should be interpreted as hypothesis‑generating and require confirmation in trials specifically designed to compare short‑ versus prolonged α_2_‑agonist–based sedation strategies. Moreover, while subgroup analyses provided valuable insights, they are subject to limitations. Specifically, findings in certain strata (e.g. ICU length of stay reduction in the sepsis-without-shock subgroup) are derived from a smaller number of trials with residual heterogeneity. Consequently, these particular subgroup effects could be influenced by lack of statistical power or the inflated risk of Type I error inherent in multiple testing, and thus warrant cautious interpretation.

The recent ADRESS pilot trial [20] illustrates this challenge: DEX was not tested as a standalone or protocol-driven sedation strategy, but rather integrated into usual care alongside conventional sedatives. While such an approach reflects real-world practice, it inevitably increases clinical heterogeneity and reduces the likelihood of detecting a specific physiological or outcome signal attributable to α_2_-agonism. In this context, the absence of a demonstrated effect should be interpreted as a limitation of trial design rather than definitive evidence of inefficacy.

## Future directions

A key explanation for the neutral findings of this meta-analysis is that DEX-based sedation represents only one element of a broader, multimodal treatment framework in critically ill patients with sepsis. Without integrated care, including spontaneous-breathing-oriented ventilatory strategies, iterative hemodynamic assessment with timely volume optimization, cautious vasopressor titration, and protocolized sedation, any potential benefit of α2-agonism may be masked by substantial background variability. This challenge is particularly evident in large multicenter trials, where DEX is often used alongside conventional sedatives rather than implemented as a distinct treatment strategy. As a result, increasing heterogeneity in institutional protocols and bedside practices can dilute treatment signals and paradoxically reduce effective statistical power, even in large samples. Dargent et al. [56] illustrates this issue, as DEX was not evaluated against a fully distinct conventional-sedation strategy, which may have attenuated any detectable α2-agonist-specific physiological effect.

Accordingly, DEX should not be viewed as intrinsically “good” or “bad” independent of clinical context. In sepsis and septic shock, treatment effects are strongly implementation-dependent: the signal of α2-agonism can be amplified or obscured by co-interventions, sedation targets, and hemodynamic and ventilatory management. Therefore, neutral findings from trials in which dexmedetomidine is used primarily as an adjunct within heterogeneous background sedation should not be interpreted as definitive evidence of pharmacologic inefficacy; rather, they may reflect a low signal-to-noise environment. The ADRESS pilot trial provides a representative example in which broad co-treatment variability may have diluted dexmedetomidine-specific effects [20].

Therefore, favorable outcomes should not be expected from dexmedetomidine or any sedation intervention, unless it is embedded in a coherent α2-based multimodal strategy: conservative titration (“start low, go slow,” generally avoiding bolus dosing), iterative assessment of preload and perfusion with timely volume optimization, minimization of competing deep-sedation exposure, and early preservation/recovery of spontaneous breathing when clinically feasible [57]. This implementation principle is consistent with prior physiology-driven reports and with broader critical care experience showing that therapeutic success depends as much on how an intervention is delivered as on the intervention itself.

## Conclusion

In mechanically ventilated patients with sepsis and septic shock, dexmedetomidine was associated with a shorter duration of mechanical ventilation but a higher risk of bradycardia, without clear benefit in mortality, SOFA score, or ICU length of stay. These pooled findings should be interpreted in the context of implementation: dexmedetomidine is highly context dependent, and favorable outcomes are unlikely to be reproducibly achieved without protocolized, multimodal α2-agonist-based management. In practice, this includes cautious titration, iterative hemodynamic optimization, minimization of deep co-sedation, and early spontaneous-breathing-oriented ventilatory management when appropriate. Future trials should prioritize high-fidelity, physiology-informed protocols with strict control of co-interventions and prespecified patient phenotypes to better distinguish true treatment effects from background practice variability.

## Supplementary Material

Supplemental Material

## Data Availability

The data that support the findings of this study are available from the corresponding author, Kai Zhang, upon reasonable request.
