# Supplementary material for: The effect of dexmedetomidine in mechanically ventilated patients with sepsis and septic shock: a meta-analysis of randomized controlled trials
Source: Ann Med. 2026 Mar 17;58(1):2643971. doi: 10.1080/07853890.2026.2643971 (PMC13003857; doi:10.1080/07853890.2026.2643971)
Supplement: Supplemental Material [file IANN_A_2643971_SM3571.zip › suppl_data/Legends for supplementary material.docx]

**Legends of supplementary materials**

**Supplementary Material 1:** PRISMA checklist

**Supplementary Material 2:** Search strategy in PubMed, Embase, Scopus and Cochrane library

**Supplementary Material 3:** Detailed intervention and control methods, sedation goals, background sedatives, baseline vasopressor dose of included studies

**Supplementary Material 4:** Publication bias assessment by funnel plot and Egger’s test, forest plot after trim and fill method

**Supplementary Material 5:** Forest plot for overall mortality at different mortality endpoints: ICU mortality, 28/30-day mortality, 90-day mortality

**Supplementary Material 6:** Results of subgroup analyses

**Supplementary Material 7:** Results of sensitivity analyses
