# Supplementary material for: The effect of dexmedetomidine in mechanically ventilated patients with sepsis and septic shock: a meta-analysis of randomized controlled trials
Source: Ann Med. 2026 Mar 17;58(1):2643971. doi: 10.1080/07853890.2026.2643971 (PMC13003857; doi:10.1080/07853890.2026.2643971)
Supplement: Supplemental Material [file IANN_A_2643971_SM3571.zip › suppl_data/Sfile 2 Search strategies.docx]

**Supplementary Material 2: Searching strategies**

**Pubmed**

#1 sepsis [MeSH Terms] OR sepsis [Title/Abstract] OR septic [Title/Abstract]

#2 dexmedetomidine [Title/Abstract] OR dexmedetomidine [MeSH Terms]

#3 randomized controlled trial [MeSH Terms] OR random* [Title/Abstract]

#1 AND #2 AND #3

**Embase**

#1 ‘sepsis’:ti,ab,kw OR ‘septic’:ti,ab,kw OR ' sepsis '/exp OR ' septic '/exp

#2 dexmedetomidine:ti,ab,kw OR ' dexmedetomidine '/exp

#3 'randomized controlled trial'/de OR 'randomized controlled trial'/exp

#1 AND #2 AND #3

**Scopus**

#1 TITLE-ABS-KEY (sepsis) OR TITLE-ABS-KEY (septic)

#2 TITLE-ABS-KEY (dexmedetomidine)

#3 TITLE-ABS-KEY (randomized) OR TITLE-ABS-KEY (random) OR TITLE-ABS-KEY (randomised)

#1 AND #2 AND #3

**Cochrane Library**

#1 (sepsis):ti,ab,kw OR (septic):ti,ab,kw

#2 (dexmedetomidine):ti,ab,kw

#3 (randomized):ti,ab,kw OR (randomised):ti,ab,kw OR (random):ti,ab,kw

#1 AND #2 AND #3
