# Supplementary material for: The effect of dexmedetomidine in mechanically ventilated patients with sepsis and septic shock: a meta-analysis of randomized controlled trials
Source: Ann Med. 2026 Mar 17;58(1):2643971. doi: 10.1080/07853890.2026.2643971 (PMC13003857; doi:10.1080/07853890.2026.2643971)
Supplement: Supplemental Material [file IANN_A_2643971_SM3571.zip › suppl_data/Sfile 3.docx]

**Supplementary Material 3： Detailed intervention and control methods, sedation goals, background sedatives, baseline vasopressor dose of included studies**

| **Study, year** | **Intervention and control methods** | **Sedation goals** | **Background sedatives** | **Vasopressor dose at baseline** |
| --- | --- | --- | --- | --- |
| Mokhlesian 2025 | Intervention: DEX start at a loading dose of 1 μg/kg·h, followed at 0.2 to 2.5 μg/kg·h, for 1 day; Control: morphine and midazolam for 0.5 to 5 mg/h | RASS score of -2 to 0 | None | NR |
| ADRESS trial 2025 | Intervention: DEX for 1 μg/kg·h, for 1 day; Control: placebo at same rate (5% glucose) | NR | Midazolam, propofol | Intervention: norepinephrine 0.9±0.5 µg/kg/min; Control: norepinephrine 0.7±0.5 µg/kg/min; |
| Iten 2025 | Intervention: DEX for 0.1 to 1.4 μg/kg·h, for 7 days; Control: propofol and midazolam determined by treating clinician | Determined by treating clinician | NR | None |
| Patidar 2024 | Intervention: DEX for 0.15 to 1.5 μg/kg·h, for 7 days; Control: propofol for 5 to 50 μg/kg·h | RASS score of -2 to 0 | None | None |
| Ezz Al-Regal 2024 | Intervention: DEX for 0.2 to 0.7 μg/kg·h, for 2 days;  Control: usual care | RASS score of -2 to 0 | Midazolam | Intervention: norepinephrine 0.78±0.23 µg/kg/min; Control: norepinephrine 0.82±0.29 µg/kg/min; |
| Elayashy 2023 | Intervention: DEX for 0.15 to 0.75 μg/kg·h, for 1 day; Control: midazolam for 1 to 5 mg/h | RASS score of -3 to -1 | None | NR |
| MENDS2 trial 2021 | Intervention: DEX for 0.15 to 1.5 μg/kg·h, for 14 days; Control: propofol for 5 to 50 μg/kg·h | RASS score of -2 to 0 | Midazolam | NR |
| Gheibi 2020 | Intervention: DEX for 0.6 μg/kg·h, for 12 h; Control: placebo at same rate (normal saline) | NR | NR | NR |
| Liu 2020 | Intervention: DEX start at a loading dose of 1 μg/kg·h, followed at 0.2 to 0.3 μg/kg·h, for 5 days; Control: propofol start at a loading dose of 1 mg/kg, followed at 1 to 3 mg/kg·h | RASS score of -2 to 0 | NR | NR |
| SPICE III trial 2019 | Intervention: DEX start at 1 μg/kg·h, followed at adjusted dose (maximum at 1.5 μg/kg·h), for 28 days; Control: propofol directed by the treating physician | RASS score of -2 to +1 | Midazolam | NR |
| DESIRE trial 2017 | Intervention group: DEX start at start at 0.1 μg/kg·h then at 0.1-0.7 μg/kg·h, for 28 days; Control group: propofol at 0-3 mg/kg/h or midazolam at 0-0.15 mg/kg/h | RASS score of -2 to 0 | Other sedatives | NR |
| MENDS trial 2010 | Intervention group: DEX at a median rate of 0.74 μg/kg·h, max does at 1.5μg/kg·h, for 5 days; Control group: lorazepam at a median rate of 3 mg/h, max does at 10 mg/h | RASS score of -2 to +1 | Midazolam, propofol | NR |
| Tasdogan 2009 | Intervention group: DEX at a loading does of 1 μg/kg for 10min, followed by 0.2-2.5 μg/kg·h, for 1 day; Control group: propofpl at a loading does of 1 mg/kg·h for 15min, followed by 1-3 mg/kg·h | RSS ≤ 2 | NR | NR |
| Memis 2009 | Intervention group: DEX at a loading does of 1 μg/kg for 10 min, followed by 0.2-2.5 μg/kg·h, for 1 day; Control group: propofpl at a loading does of 1 mg/kg·h for 15min, followed by 1-3 mg/kg·h | RSS ≤ 2 | None | NR |
| Memis 2007 | Intervention group: DEX at a loading does of 1 μg/kg for 10 min, followed by 0.2-2.5 μg/kg·h, for 1 day; Control group: midazolam at a loading does of 0.2 mg/kg·h for 10 min, followed by 0.1-0.5 mg/kg·h | RSS < 2 | None | NR |
