# Supplementary material for: The effect of dexmedetomidine in mechanically ventilated patients with sepsis and septic shock: a meta-analysis of randomized controlled trials
Source: Ann Med. 2026 Mar 17;58(1):2643971. doi: 10.1080/07853890.2026.2643971 (PMC13003857; doi:10.1080/07853890.2026.2643971)
Supplement: Supplemental Material [file IANN_A_2643971_SM3571.zip › suppl_data/Sfile4 publication.docx]

**Supplementary Material 3:** Publication bias assessment by funnel plot and Egger’s test, forest plot after trim and fill method


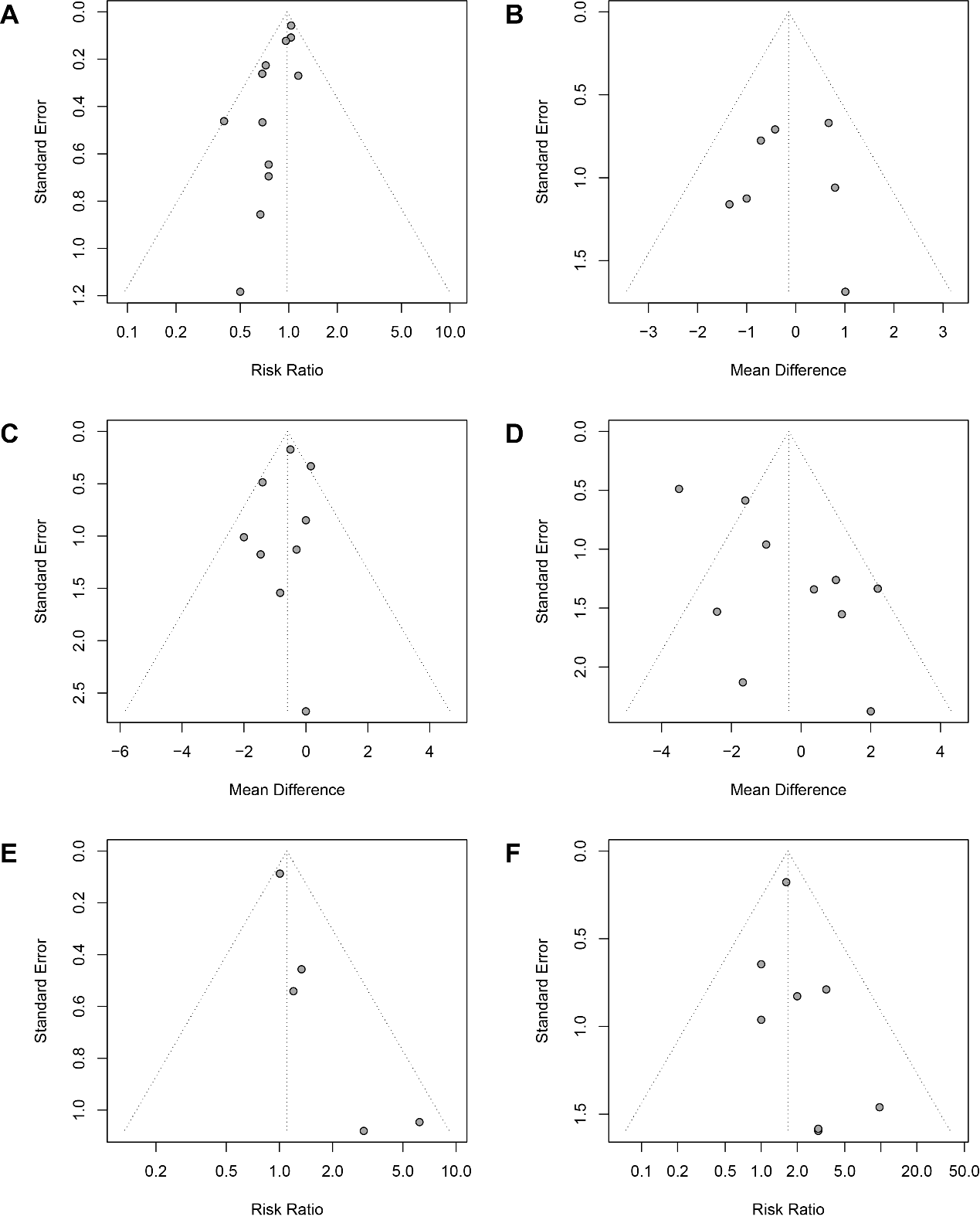


Figure 1: Funnel plot for (A) overall mortality, Egger’s test P=0.0083; (B) SOFA score; (C) duration of MV; (D) length of stay in ICU, Egger’s test P=0.0080; (E) hypotension; (F) bradycardia


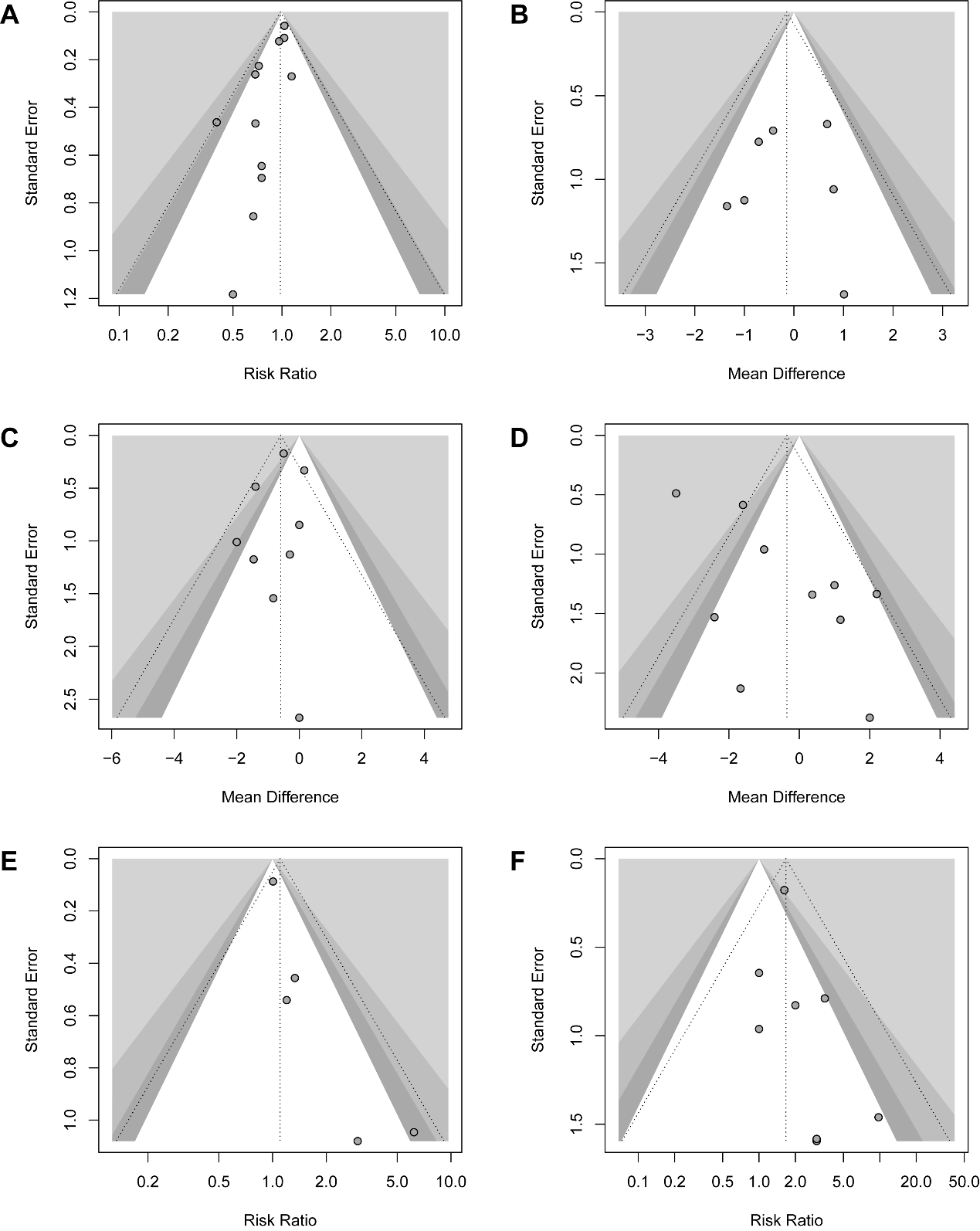


Figure 2: Contour-enhanced funnel plots for (A) overall mortality; (B) SOFA score; (C) duration of MV; (D) length of stay in ICU; (E) hypotension; (F) bradycardia


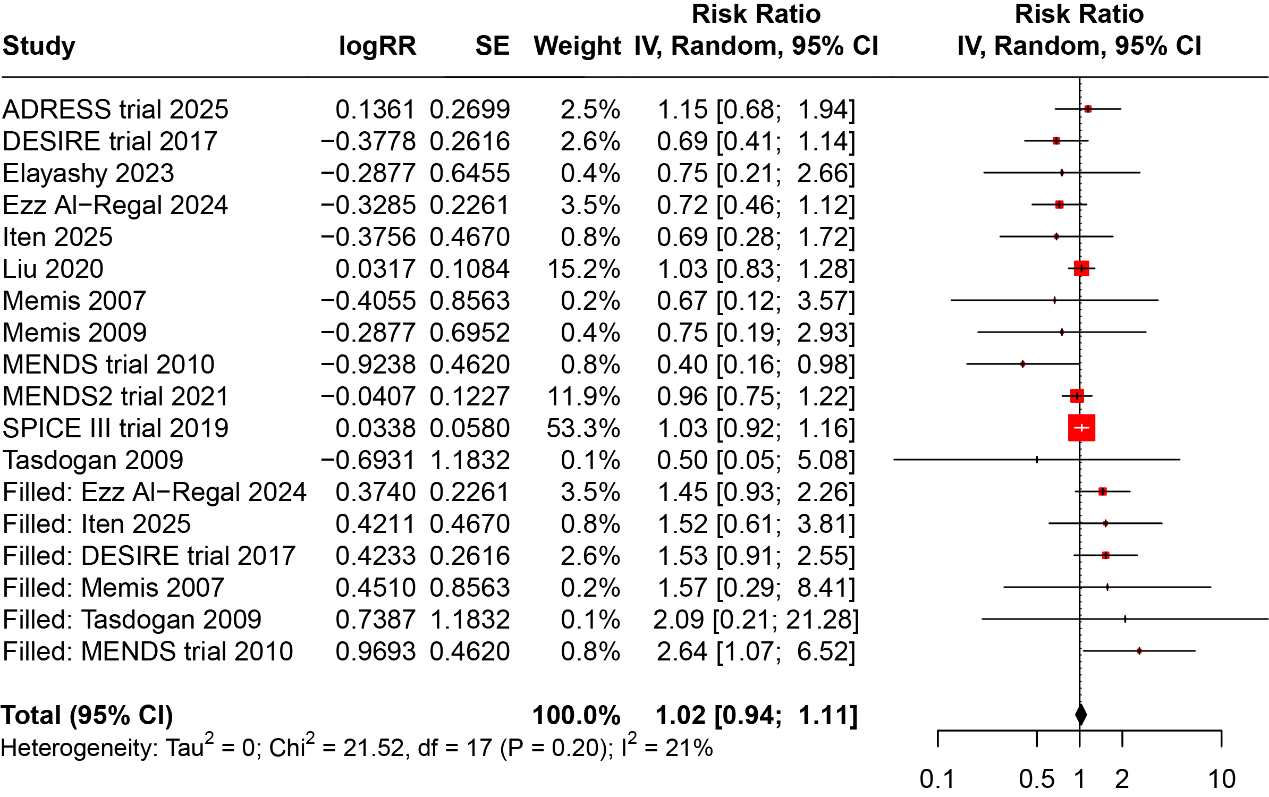


Figure 3: Forest plot for overall mortality after trim and fill method


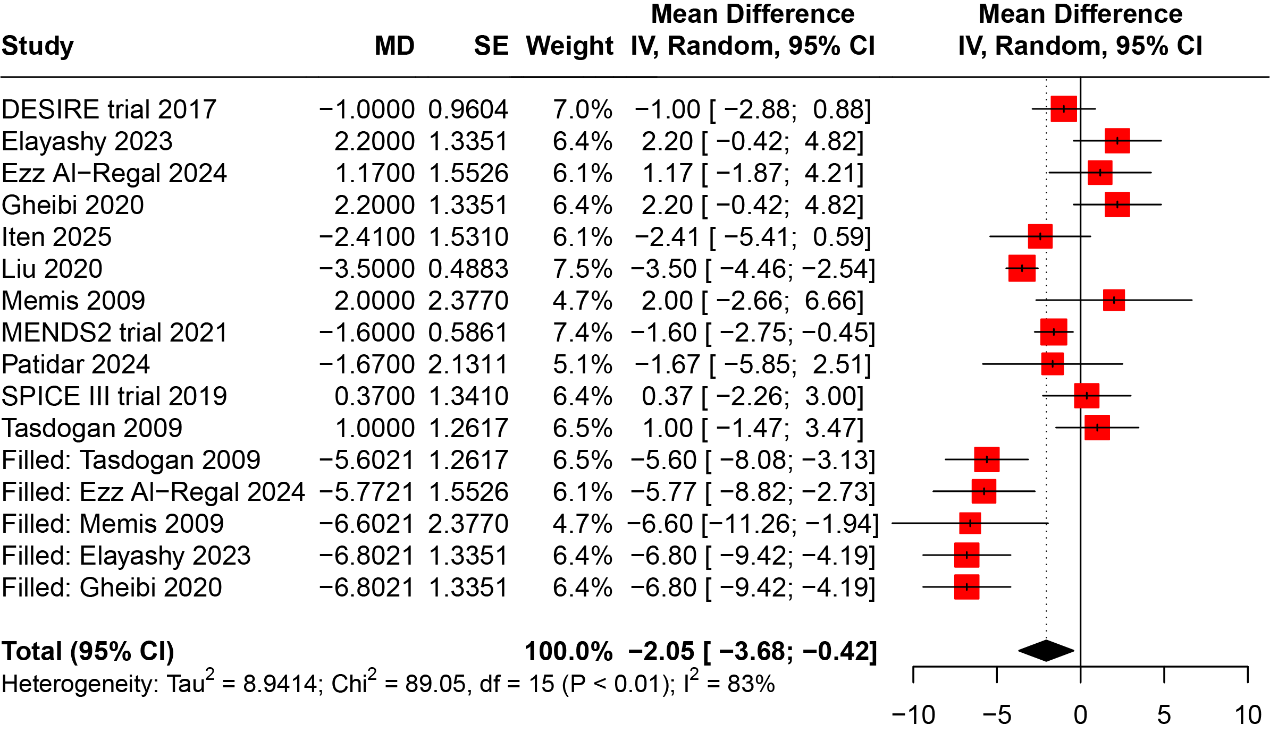


Figure 4: Forest plot for length of stay in ICU after trim and fill method


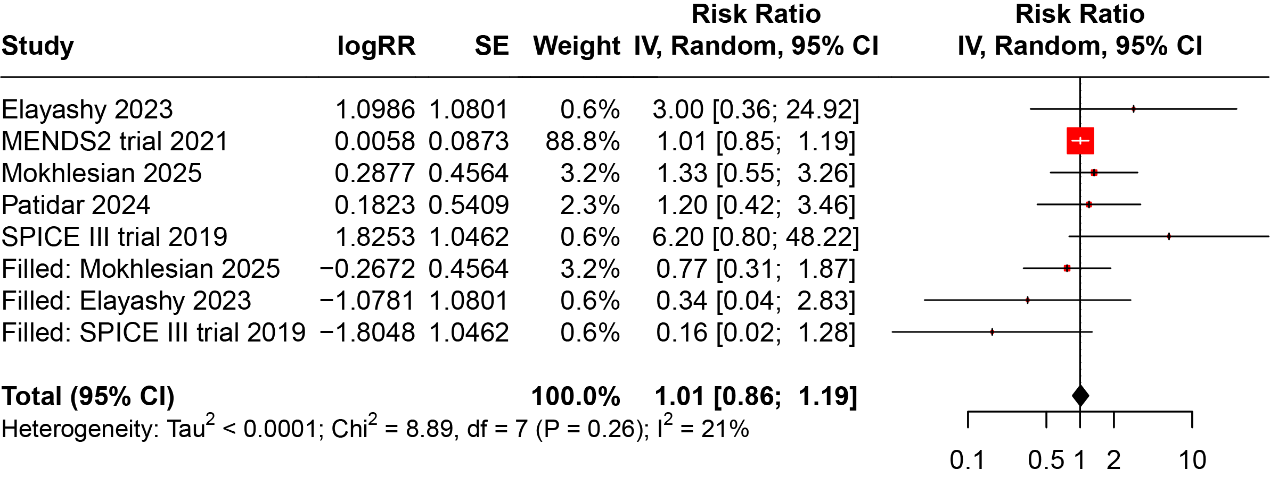


Figure 5: Forest plot for hypotension after trim and fill method


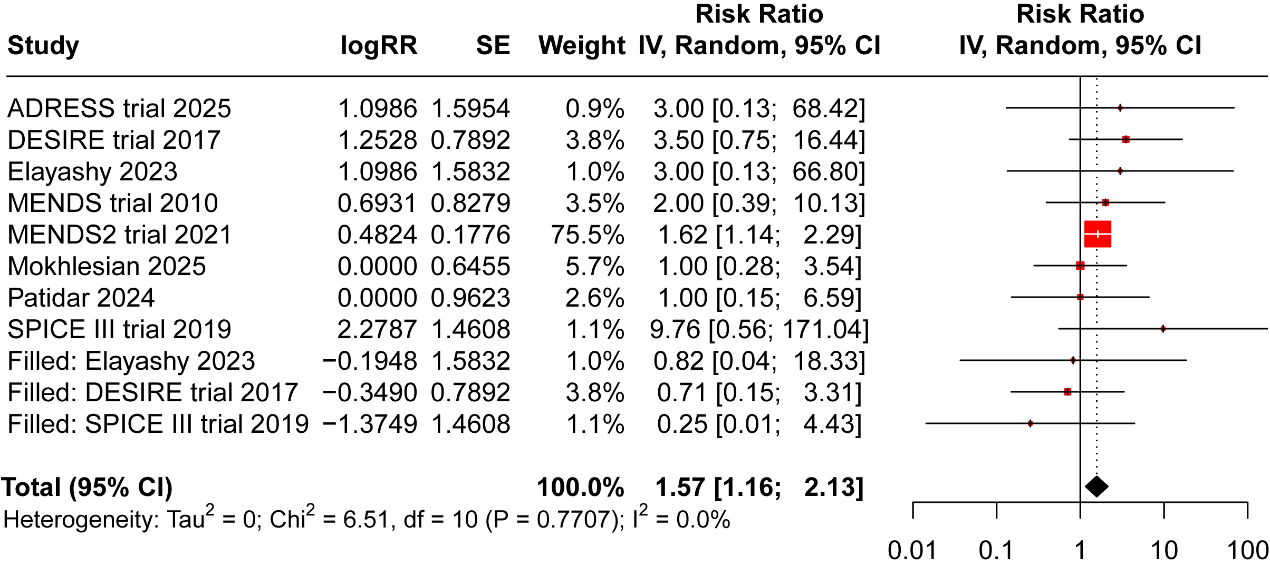


Figure 6: Forest plot for bradycardia after trim and fill method
