# Supplementary material for: The effect of dexmedetomidine in mechanically ventilated patients with sepsis and septic shock: a meta-analysis of randomized controlled trials
Source: Ann Med. 2026 Mar 17;58(1):2643971. doi: 10.1080/07853890.2026.2643971 (PMC13003857; doi:10.1080/07853890.2026.2643971)
Supplement: Supplemental Material [file IANN_A_2643971_SM3571.zip › suppl_data/Sfile6 sub.docx]

**Supplementary Material 6: Results of subgroup analyses**


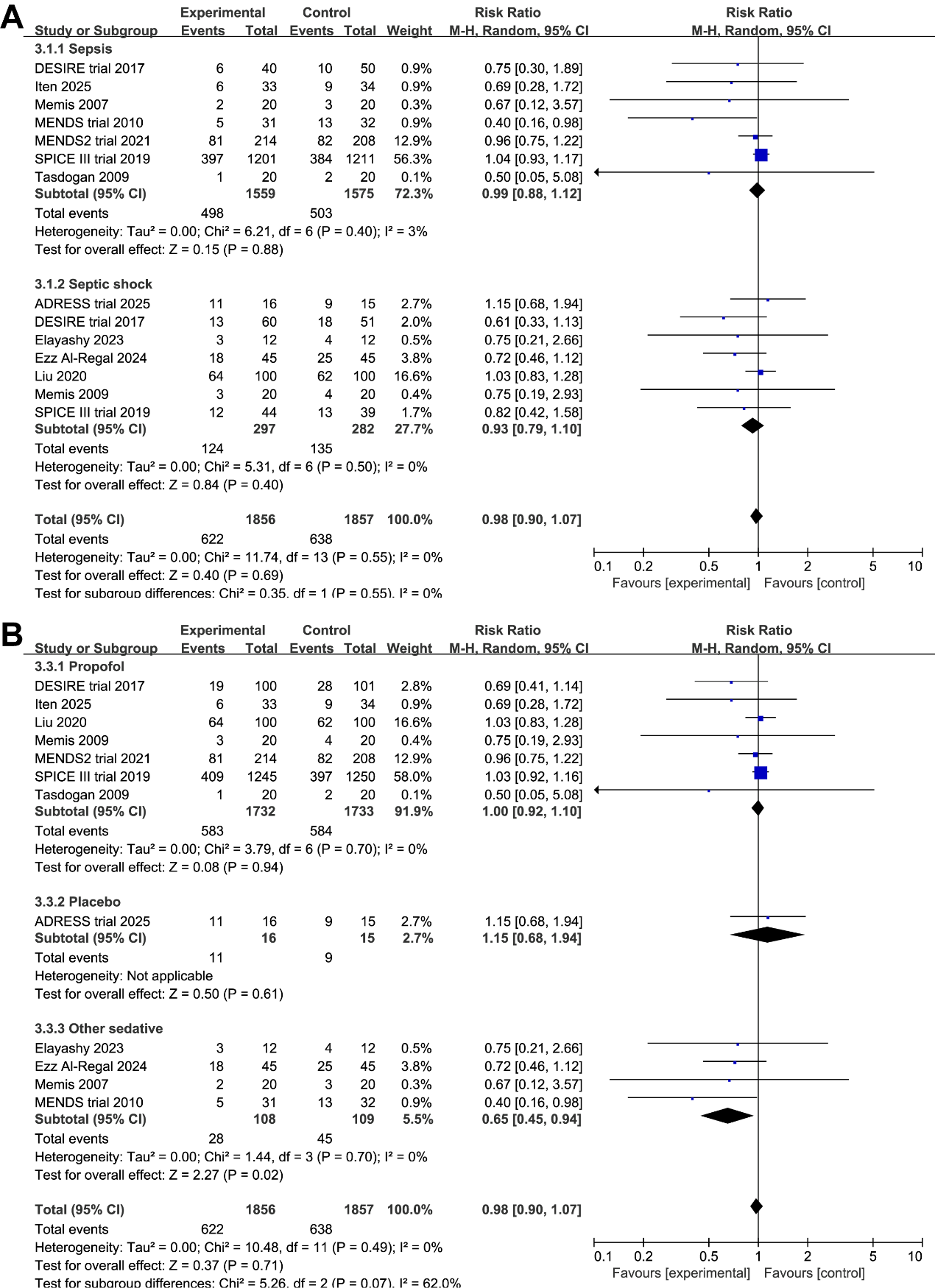


Figure 1: Subgroup analysis for overall mortality, (A) sepsis and septic shock subgroup, (B) propofol, placebo and other sedative subgroup


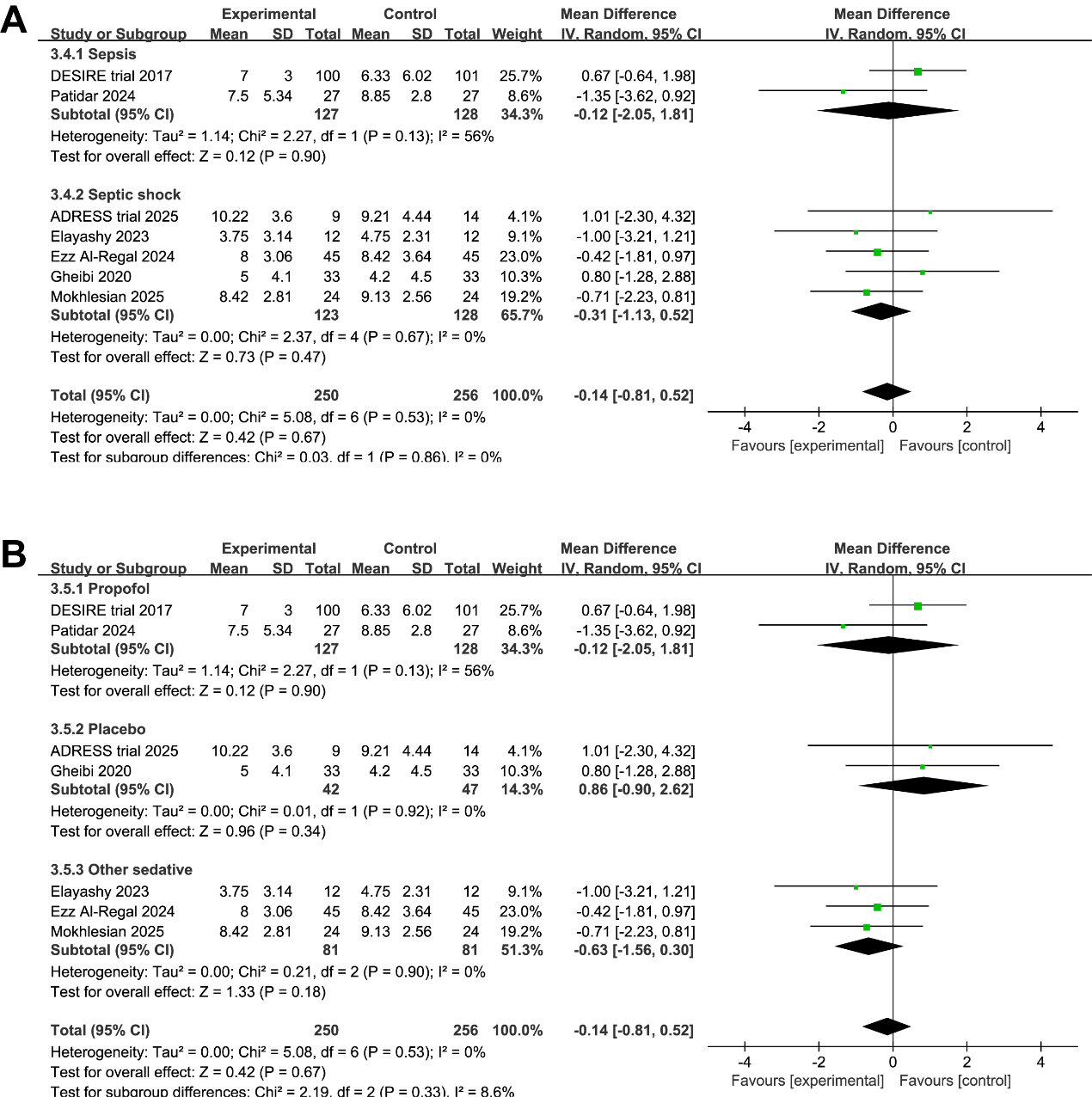


Figure 2: Subgroup analysis for SOFA score, (A) sepsis and septic shock subgroup, (B) propofol, placebo and other sedative subgroup


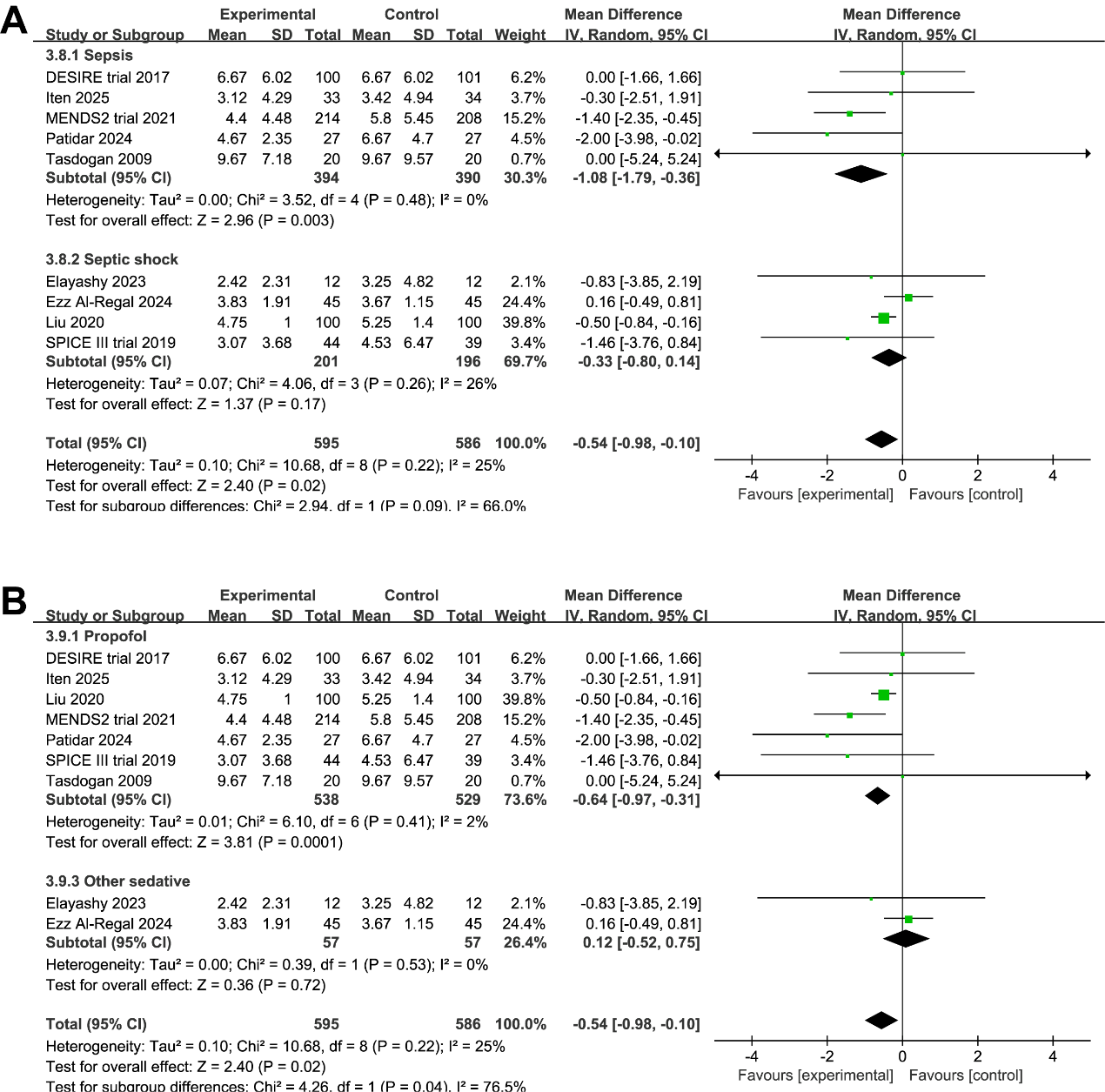


Figure 3: Subgroup analysis for duration of MV, (A) sepsis and septic shock subgroup, (B) propofol, placebo and other sedative subgroup


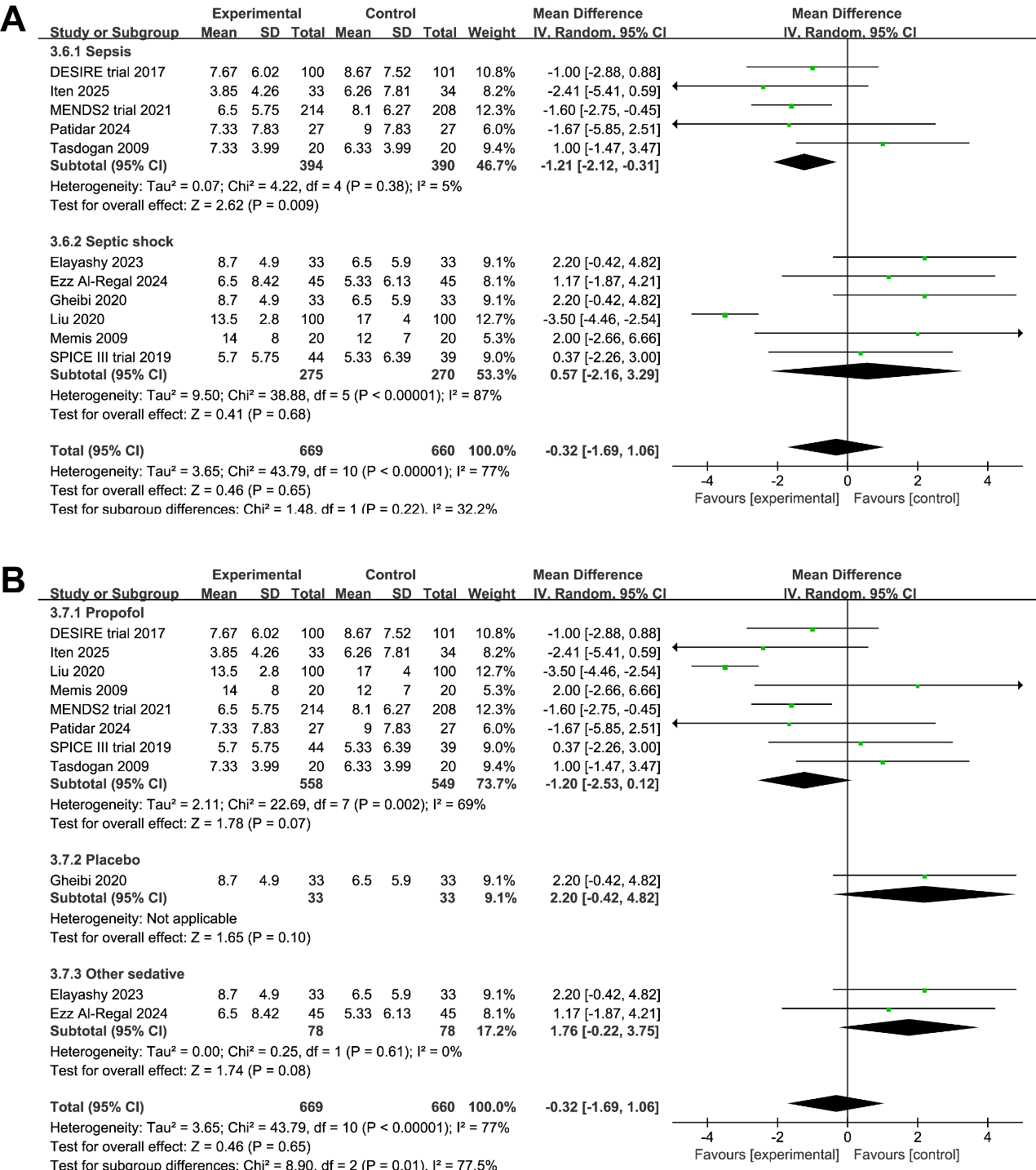


Figure 4: Subgroup analysis for length of stay in ICU, (A) sepsis and septic shock subgroup, (B) propofol, placebo and other sedative subgroup


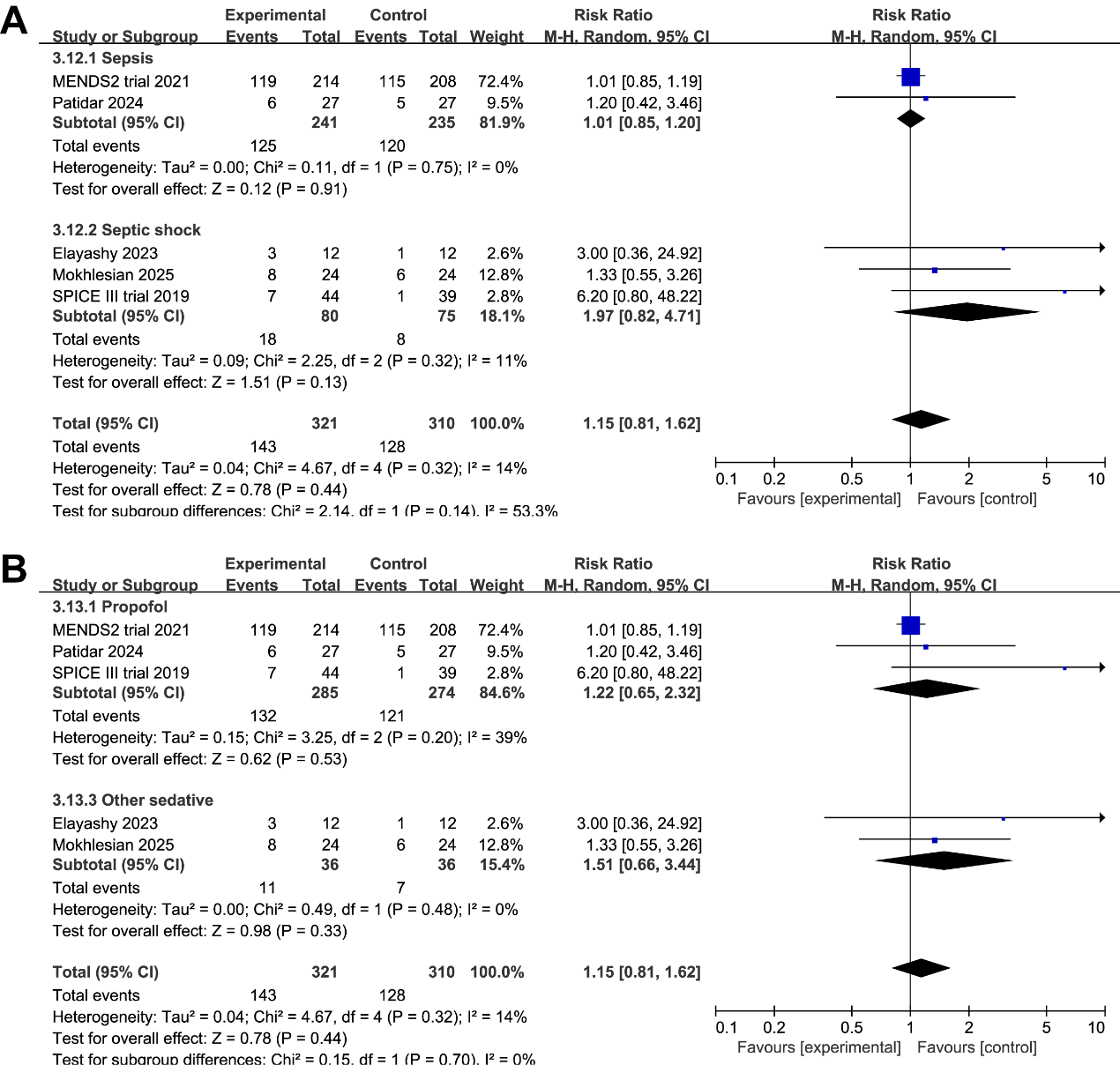


Figure 5: Subgroup analysis for hypotension, (A) sepsis and septic shock subgroup, (B) propofol, placebo and other sedative subgroup


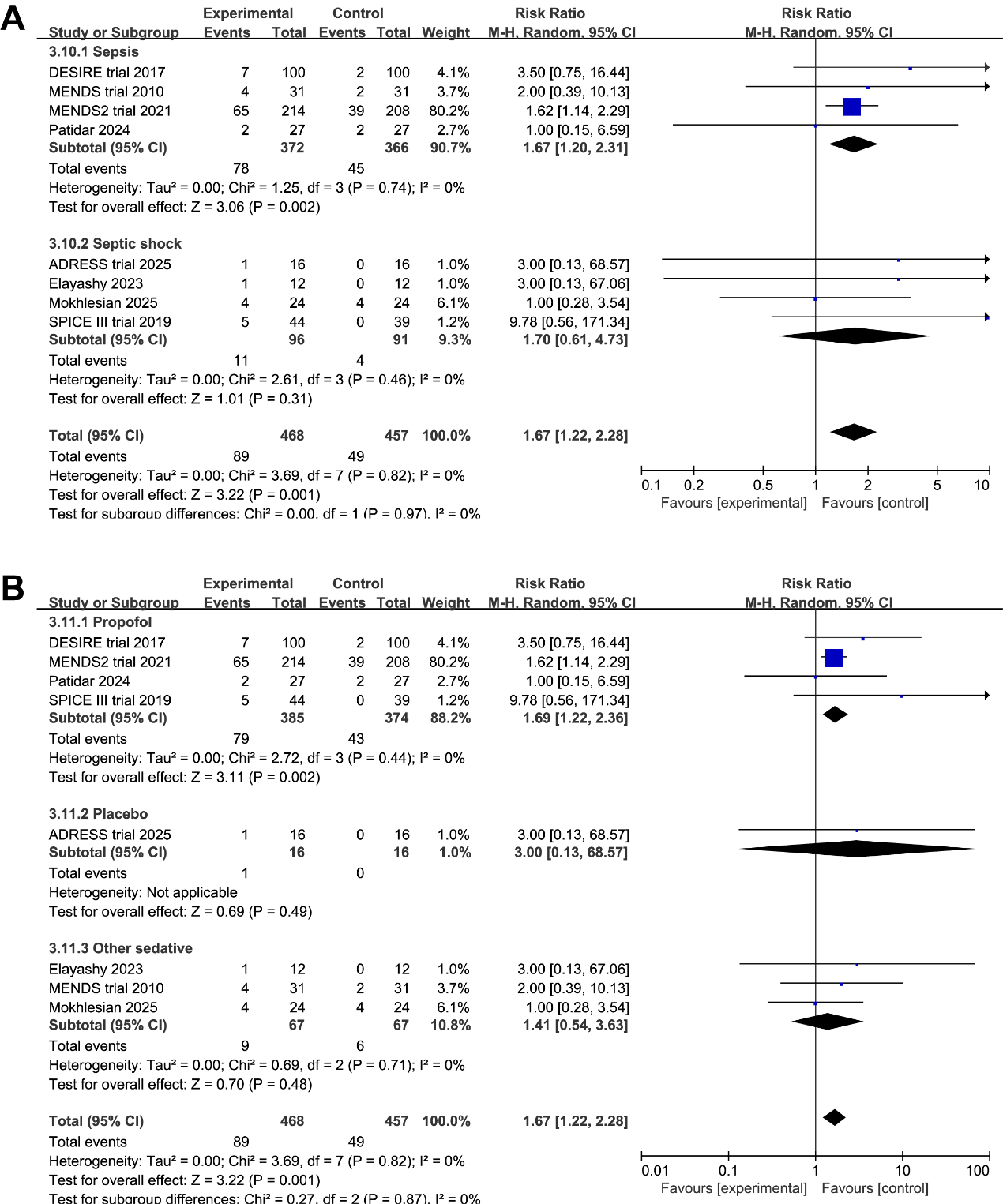


Figure 6: Subgroup analysis for bradycardia, (A) sepsis and septic shock subgroup, (B) propofol, placebo and other sedative subgroup


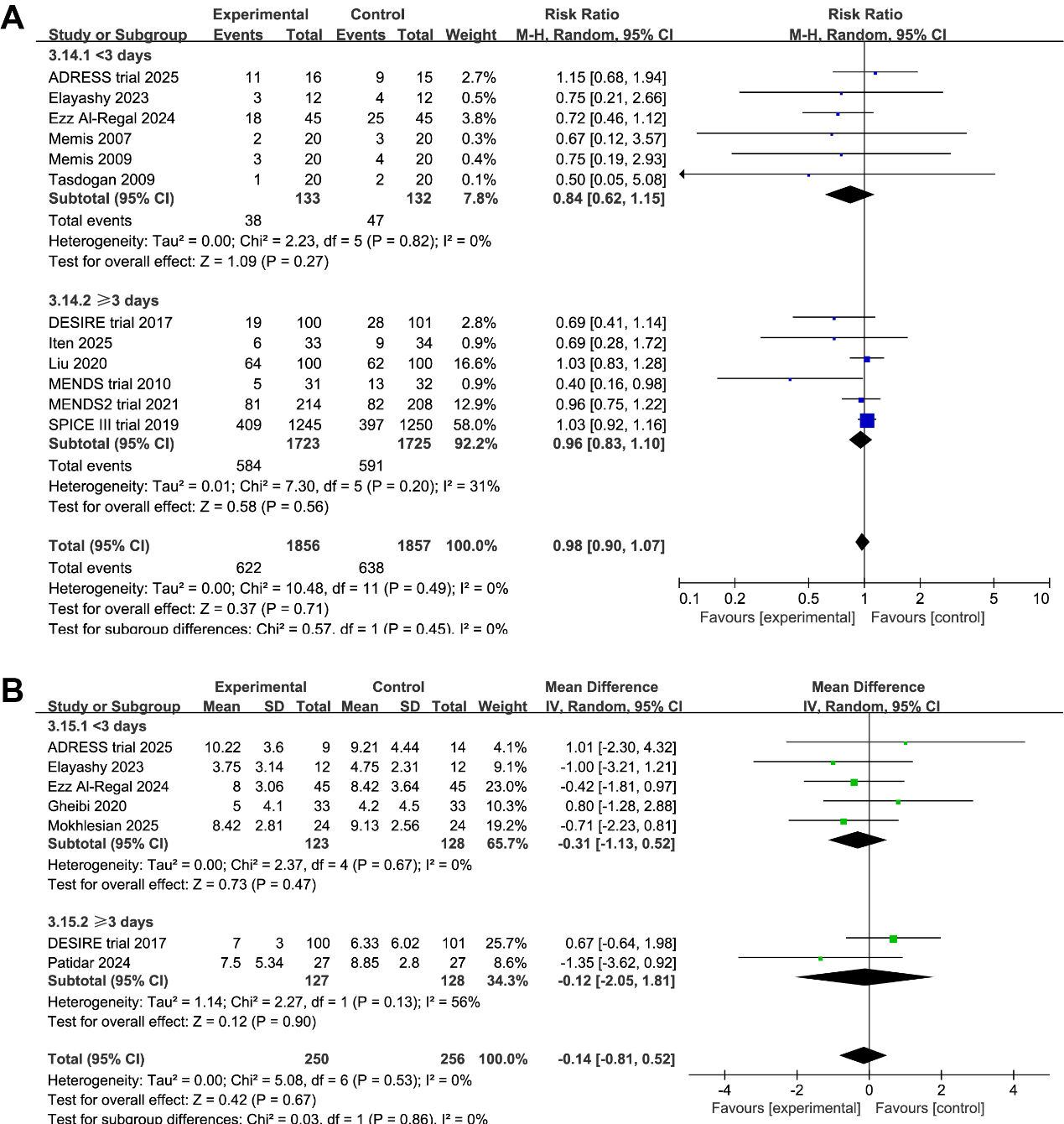


Figure 7: Post‑hoc subgroup analysis stratified by duration of intervention for (A) overall mortality, (B) SOFA score


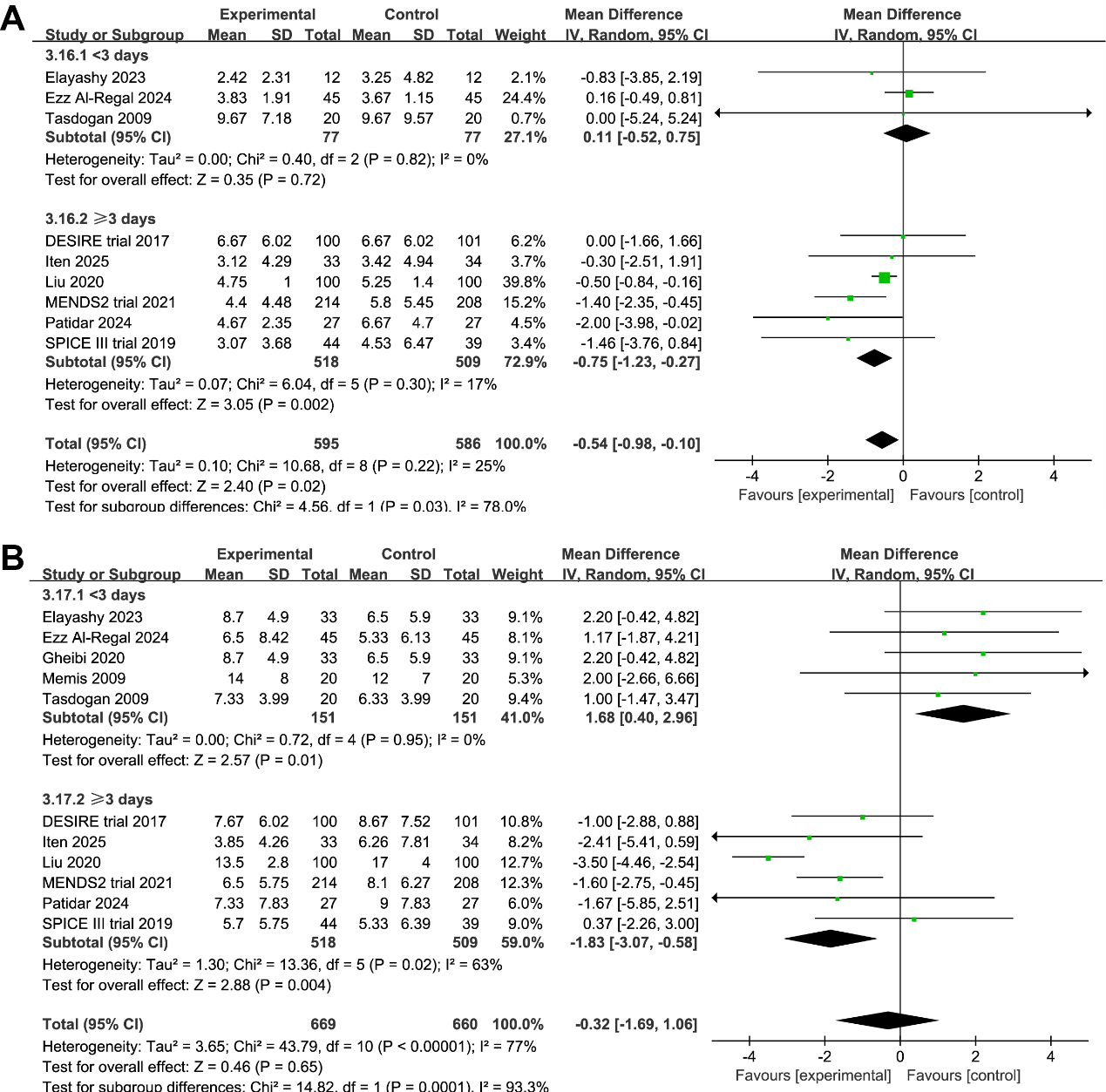


Figure 8: Post‑hoc subgroup analysis stratified by duration of intervention for (A) duration of MV, (B) length of stay in ICU


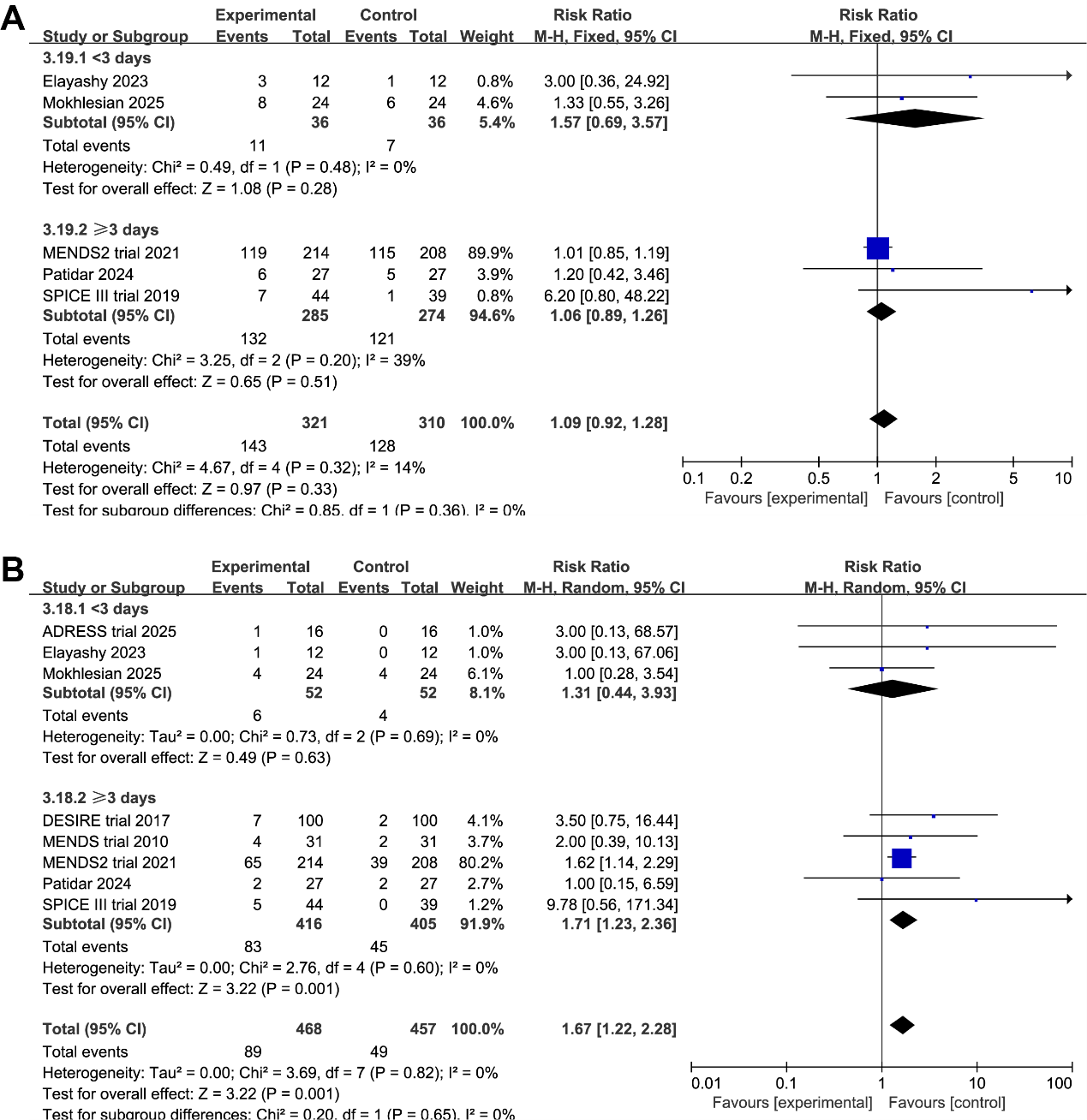


Figure 9: Post‑hoc subgroup analysis stratified by duration of intervention for (A) hypotension, (B) bradycardia
