# Supplementary material for: The effect of dexmedetomidine in mechanically ventilated patients with sepsis and septic shock: a meta-analysis of randomized controlled trials
Source: Ann Med. 2026 Mar 17;58(1):2643971. doi: 10.1080/07853890.2026.2643971 (PMC13003857; doi:10.1080/07853890.2026.2643971)
Supplement: Supplemental Material [file IANN_A_2643971_SM3571.zip › suppl_data/Sfile7 sen.docx]

**Supplementary Material 7: Results of sensitivity analyses**

**Table 1: Results of sensitivity analyses**

| **Analysis Type** | **Description** | **Pooled Effect, I^2^** |
| --- | --- | --- |
| **Mortality** |  |  |
| Primary analysis | DerSimonian-Laird random-effects model | 0.98 (0.90, 1.07), 0% |
| Heterogeneity Estimation (REML) | Restricted Maximum Likelihood for τ² | 0.98 (0.89, 1.07), 0% |
| Exclusion of High RoB Studies | Excluded studies with a high risk of bias in any domain | 0.90 (0.76, 1.06), 2% |
|  |  |  |
| **SOFA** |  |  |
| Primary analysis | DerSimonian-Laird random-effects model | -0.14 (-0.81, 0.52), 0% |
| Heterogeneity Estimation (REML) | Restricted Maximum Likelihood for τ² | -0.14 (-0.81, 0.52), 0% |
| Exclusion of Median-Conv. Studies | Included only studies reporting mean ± SD | -0.31 (-1.33, 0.72), 0% |
| Exclusion of High RoB Studies | Excluded studies with a high risk of bias in any domain | -0.14 (-0.81, 0.52), 0% |
|  |  |  |
| **Duration of MV** |  |  |
| Primary analysis | DerSimonian-Laird random-effects model | -0.54 (-0.98, -0.10), 25% |
| Heterogeneity Estimation (REML) | Restricted Maximum Likelihood for τ² | -0.59 (-1.14, -0.04), 25% |
| Exclusion of Median-Conv. Studies | Included only studies reporting mean ± SD | -1.41 (-2.29, -0.53), 0% |
| Quantile Estimation (QE) | Median-based approach for skewed data | -0.89 (-1.78, -0.01), 0% |
| Exclusion of High RoB Studies | Excluded studies with a high risk of bias in any domain | -1.10 (-1.76, -0.43), 0% |
|  |  |  |
| **Length of ICU stay** |  |  |
| Primary analysis | DerSimonian-Laird random-effects model | -0.32 (-1.69, 1.06), 77% |
| Heterogeneity Estimation (REML) | Restricted Maximum Likelihood for τ² | -0.35 (-1.64, 0.94), 77% |
| Exclusion of Median-Conv. Studies | Included only studies reporting mean ± SD | -0.20 (-1.99, 1.60), 61% |
| Quantile Estimation (QE) | Median-based approach for skewed data | -0.55 (-1.45, 0.35), 0% |
| Exclusion of High RoB Studies | Excluded studies with a high risk of bias in any domain | -0.04 (-1.24, 1.16), 52% |
|  |  |  |
| **Bradycardia** |  |  |
| Primary analysis | DerSimonian-Laird random-effects model | 1.67 (1.22, 2.28), 0% |
| Heterogeneity Estimation (REML) | Restricted Maximum Likelihood for τ² | 1.67 (1.22, 2.28), 0% |
| Exclusion of High RoB Studies | Excluded studies with a high risk of bias in any domain | 1.67 (1.22, 2.28), 0% |
|  |  |  |
| **Hypotention** |  |  |
| Primary analysis | DerSimonian-Laird random-effects model | 1.15 (0.81, 1.62), 14% |
| Statistical Method |  |  |
| Heterogeneity Estimation (REML) | Restricted Maximum Likelihood for τ² | 1.10 (0.83, 1.46), 9% |
| Exclusion of High RoB Studies | Excluded studies with a high risk of bias in any domain | 1.15 (0.81, 1.62), 14% |


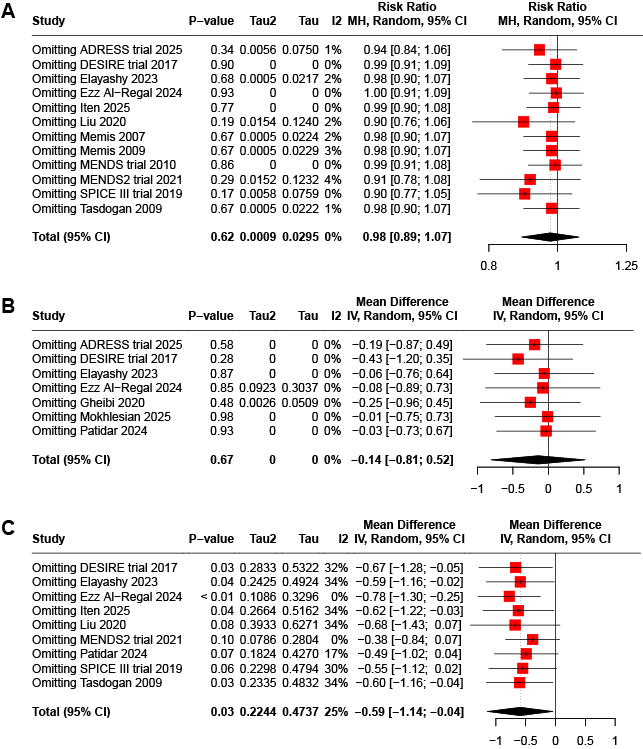


Figure 1: Forest plots for sensitivity analyses through sequential exclusion methods, (A) overall mortality, (B) SOFA score, (C) duration of MV


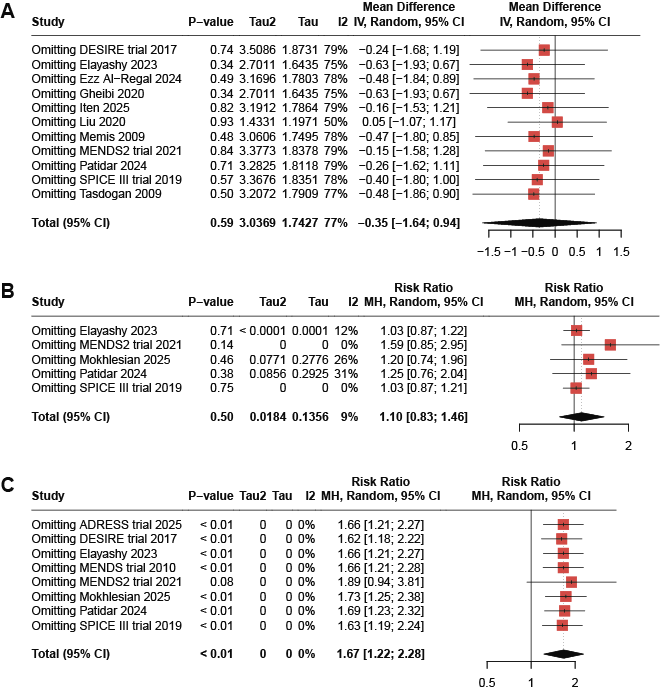


Figure 2: Forest plots for sensitivity analyses through sequential exclusion methods, (A) length of stay in ICU, (B) hypotension, (C) bradycardia
