# Supplementary figures and images for: The effect of dexmedetomidine in mechanically ventilated patients with sepsis and septic shock: a meta-analysis of randomized controlled trials
Source: Ann Med. 2026 Mar 17;58(1):2643971. doi: 10.1080/07853890.2026.2643971 (PMC13003857; doi:10.1080/07853890.2026.2643971)

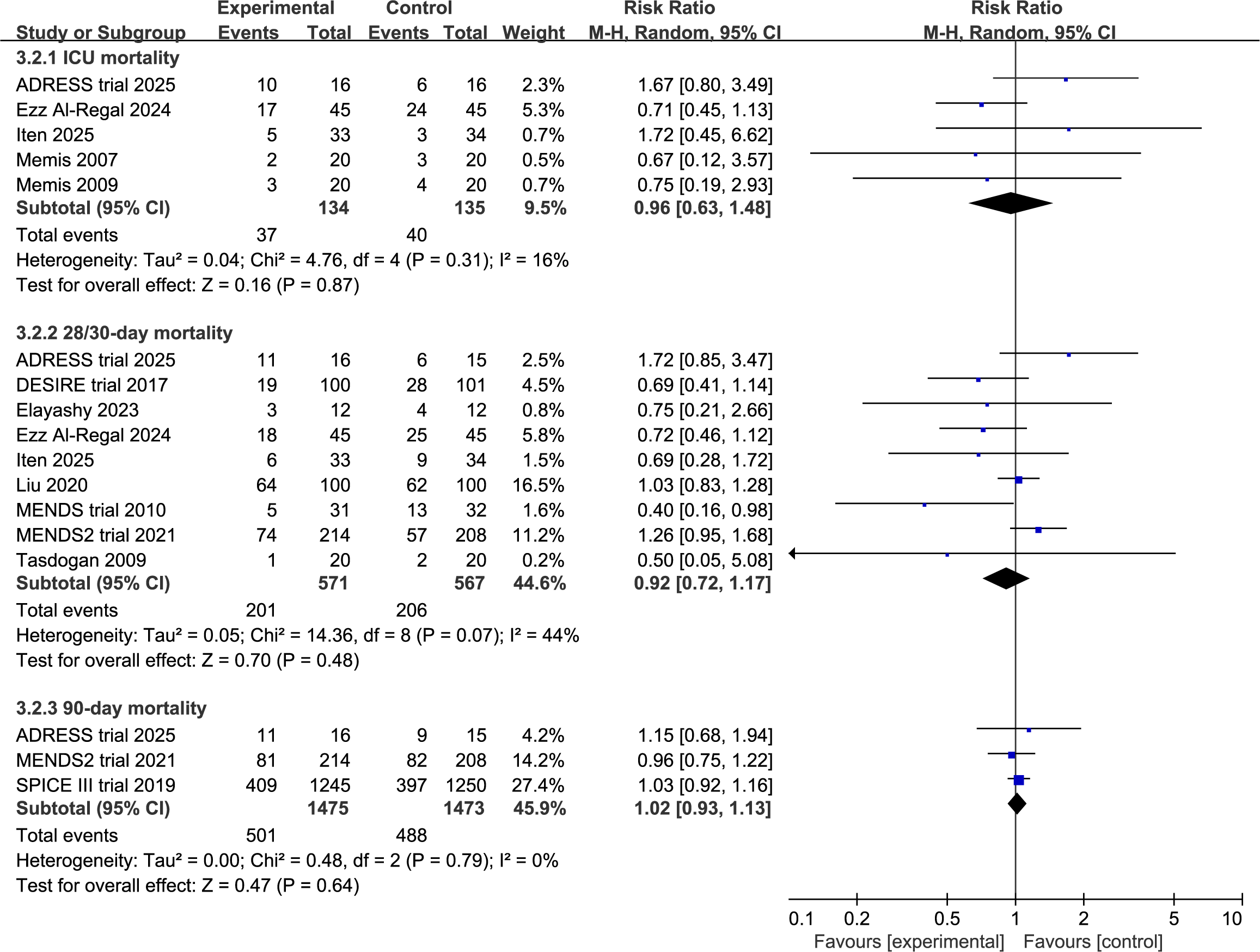

Supplement: Supplemental Material [file IANN_A_2643971_SM3571.zip › suppl_data/sfile5 mortime.tif]
